# MyChemise: A 2D drawing program that uses morphing for visualisation purposes

**DOI:** 10.1186/1758-2946-3-53

**Published:** 2011-12-12

**Authors:** Jörg-Hubertus Wilhelm

**Affiliations:** 1 31275 Lehrte, Niedersachsen, Germany

## Abstract

MyChemise (My Chemical Structure Editor) is a new 2D structure editor. It is designed as a Java applet that enables the direct creation of structures in the Internet using a web browser. MyChemise saves files in a digital format (.cse) and the import and export of .mol files using the appropriate connection tables is also possible.

MyChemise is available as a free online version in English and German. The MyChemise GUI is designed to be user friendly and can be used intuitively. There is also an English and German program description available as a PDF file.

In addition to the known ways of drawing chemical structure formulas, there are also parts implemented in the program that allow the creation of different types of presentation. The morphing module uses this technology as a component for dynamic visualisation. For example, it enables a clear and simple illustration of molecule vibrations and reaction sequences.

## Introduction

2D drawing programs account for some of the first computer applications in chemistry and are widely distributed. Many publications, especially in recent times [1-3], show that this sector is developing continually. The incorporation of new programming languages or improvements in methods for targeted structure searching in the Web continues to present a challenge for chemists who are interested in programming.

Having been given the option at work of setting up a new database with conventional structural images, the author asked himself the question how he could get hold of these images. Four options were considered:

1. To anchor the images using a link into the Web (e.g. using CAS-numbers).

2. Copy the structural images from a source in the Web.

3. Use a program already available on the market for drawing.

4. Develop a program oneself.

The first two options can be ruled out because not all the compounds entered were available as finished structures. Additionally, the structural images should look standardised if they are to be used as a marketing instrument for customer relations. Obtaining the images from different sources would have been unsatisfactory because the sizes and types of representations vary greatly. Known drawing programs would have been useable; however the drawings would have still required subsequent work in order to achieve a company specific layout. Therefore the fourth option seemed most sensible and the most interesting.

MyChemise was written with the intention of producing a stand-alone approach in this field and because of the fun in programming chemistry software. The Version 11.01 presented here was created as sideline work in the period between January 2008 and March 2011.

## Implementation

MyChemise is a modern scientific online 2D drawing program for chemical structural image. It was programmed in Java and runs as an applet in any browser that has an up-to-date Java plug-in (see http://www.java.com/en/download/installed.jsp to check if one is available). MyChemise can be opened as the English version http://www.knalltundstinkt.de/MyChemise_englisch/ChemiseZert_Home.html. If MyChemise is not running it is possibly necessary to activate javascript and/or to reduce the security settings of the browser. A program description is available as a PDF file http://www.knalltundstinkt.de/MyChemise_englisch/Description.pdf and additional file 1). A German version can be accessed from the author's homepage [4] including appropriate instructions. From here you can always open the latest version. One advantage of this online technology is that any subsequent program enhancements do not need to be downloaded and there is even no need for a new installation to be carried out because the latest MyChemise version is always available online.

The readers of this article can also download a zip-file (additional file 2) if they want to install MyChemise on their desktop PC. It contains two files (MyChemise.html and ChemJar.jar). If you unzip both into the same folder it is possible to start MyChemise in your browser (with up-to-date Java plug-in) with a double-click on MyChemise.html (in case of Windows platforms) even in the off-line mode, too.

MyChemise is a signed applet, which means that it does not have to obey the applet sandbox principle intended for security purposes. It is possible to work online in the browser and once the work is finished to save the files on your own PC. This means that drawings can be directly exported as image files into other applications using the clipboard (Note for Linux users: see program description).

The (theoretical) possible number of atoms that can be drawn in a file in MyChemise was set to 100000.

MyChemise is optimized for Windows platforms with Firefox as browser. The minimum system requirements are a 1.6 GHz processor and 2 GB of RAM.

## Results and discussion

**Figure 1:**
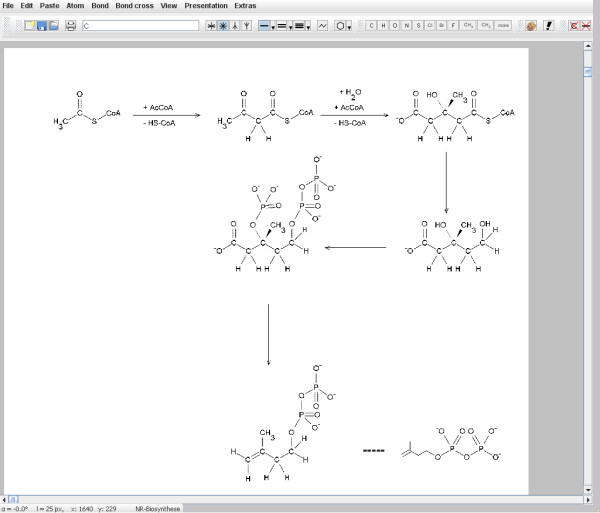
**Input screen**. It shows the menu items and the file-toolbar.

The input screen (Figure 1) shows the most important commands, relevant menu items and toolbars in a clear way. This means that the screen is not overloaded with symbols belonging to windows program technology, only those toolbars that belong to the menu items that have just been opened are made visible.

All atoms and bonds can be coloured. This allows aesthetically pleasing structural images to be easily created. Formulae can be represented in long form (with C- and H atoms labelled) and in short form. Many different bond types are available for selection. Cyclic hydrocarbons and aromatics can be quickly designed from a pull-down menu. Different 6-ring conformations can be constructed in the same way. Heterocyclics can be created by simply replacing the C atoms. Bond angles and lengths can be continuously adjusted by dragging the mouse or fit to the grid. Preset molecular geometries (tetrahedral, octahedral, etc.) make drawing easier. Newman projections are also possible.

**Figure 2:**
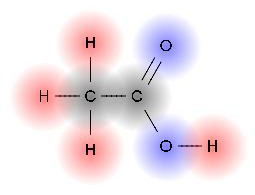
**Structural image with atomic shells**. Atomic shells can be rendered with colour gradients.

Atoms can be shown with symbolised atomic shells (Figure 2), which can be separately shaped.

**Figure 3:**
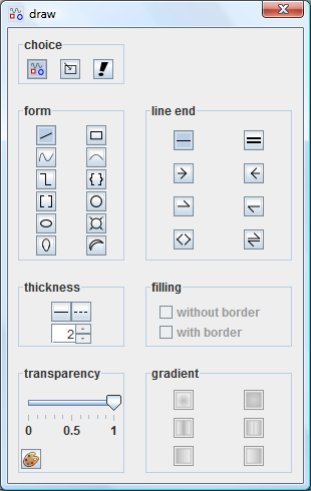
**Dialog window of drawing components**. A choice of shapes can be added to the sketch area. Some of them are useful to symbolize orbitals.

A diverse range of drawing components (arrows, brackets, shapes) can be created using a dialog window (Figure 3). Using club and banana basic shapes orbitals can be symbolically represented. The switching on of colour gradients increases this impression.

**Figure 4:**
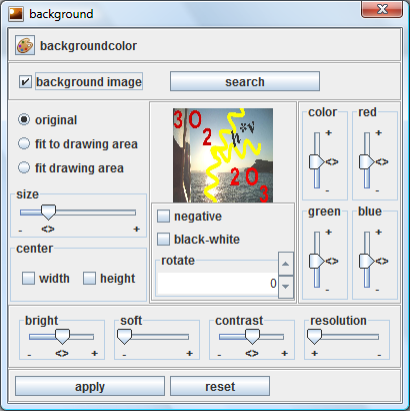
**Dialog window for the background**. It allows you to modify background images in several ways. Watermarks can be generated by increasing the brightness.

**Figure 5:**
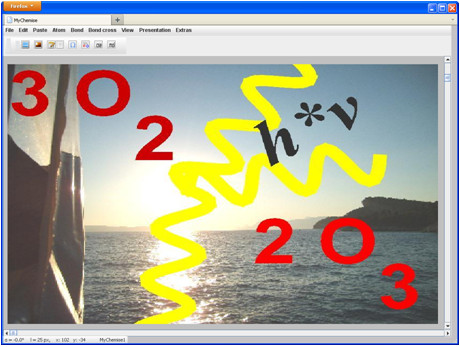
**Example for a chemical depiction with a background image**. The background image can be used as drawing surface.

**Figure 6:**
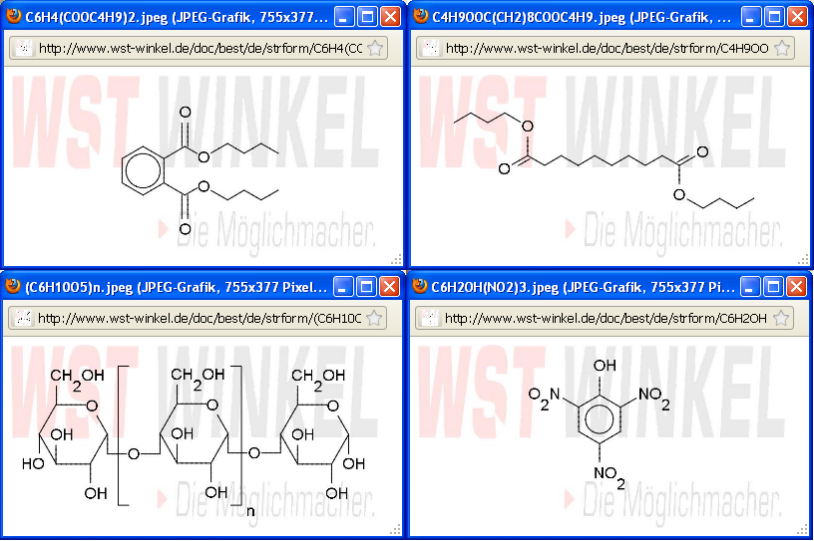
**Structural images with a company logo**. Chemical depictions with equal company logo can be used as a marketing instrument to increase corporate identity.

If you want to give the structural images an individual style, then you can insert a background image into the drawing area which can be modified using a dialog window (Figure 4 and 5). A company logo (example of use see: [5]) can be used as a marketing instrument to increase corporate identity (Figure 6).

**Figure 7:**
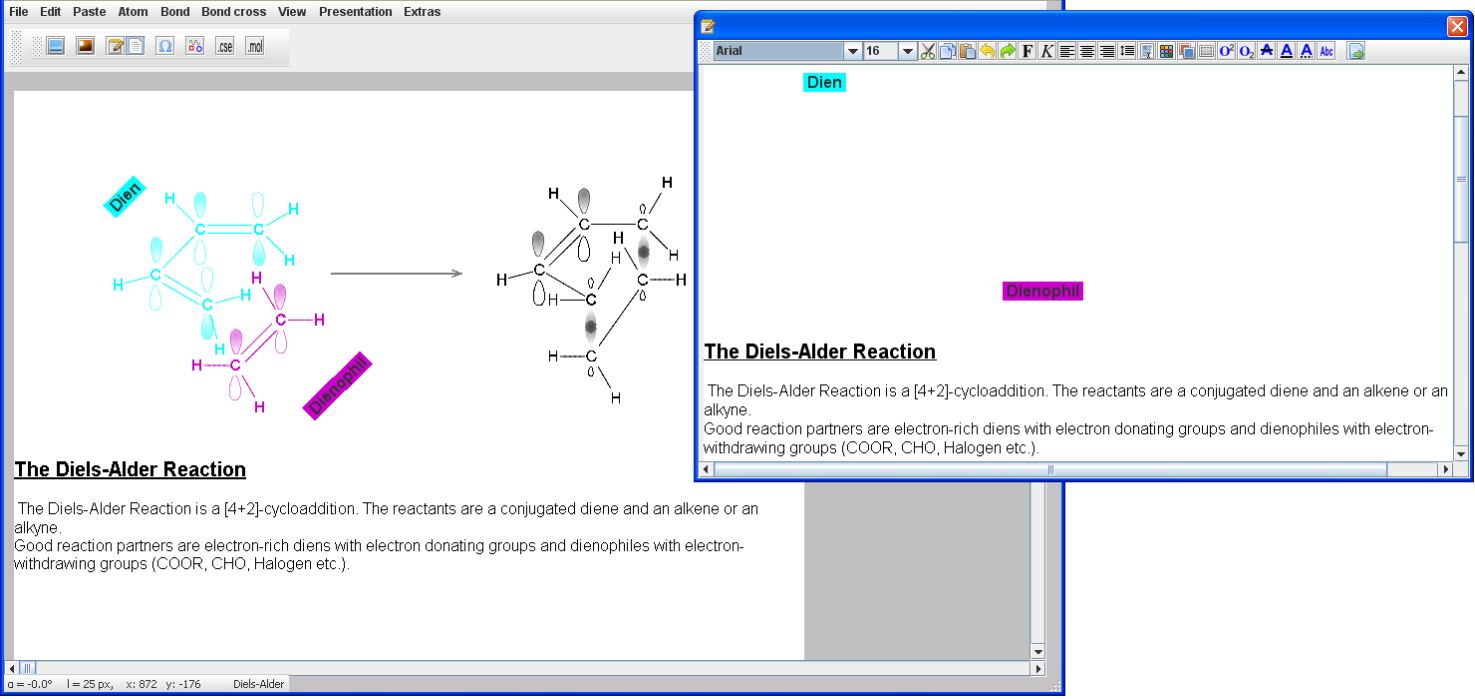
**The text editor**. With the text editor multiple rows of text can be formatted and added to the sketch area. Rotated text is only shown there.

Single rows of text can be entered using the atom input box. A special editor (Figure 7) is available for multiple rows of text. You can also use the editor to directly help you create short chemical-specific text in MyChemise, without the need for an extra writing program. This makes work easier and can help to save time, and therefore costs.

**Figure 8:**
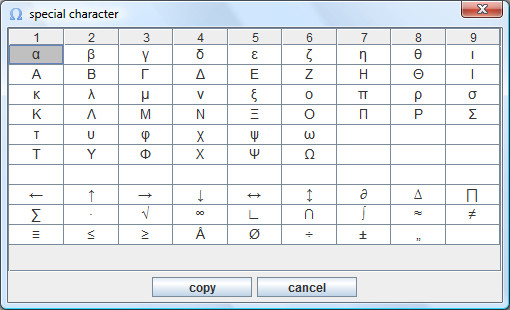
**Dialog window for special characters**. A selection of often used symbols.

A selection of special characters can be opened using a dialog window (Figure 8). Individual characters can be inserted into the editor or the atom input box by copying and pasting.

**Figure 9:**
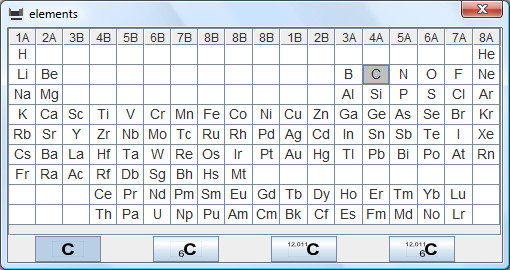
**Atomic symbols**. Atomic symbols can be pasted with their atomic and mass numbers or without.

Atomic symbols are also shown with their atomic and mass numbers (Figure 9).

### File import and export

The mol file format [6,7], version V2000 was chosen as an interface so that MyChemise can also exchange files with similar programs. Mol files can be opened in the MyChemise screen and can be saved in mol format. In addition, mol files can be inserted just by a single click of the mouse into cse files and can also be attached onto existing structures. When doing this, the atom farthest to the left is always used as the coupling atom (atom with the smallest x-coordinate).

The export function does not automatically limit the number of atoms and bonds that are to be exported. MyChemise allows the input of a large number of atoms, whilst mol files are setup for a maximum of 999 atoms and 999 bonds, this means that every user must ensure for themselves that when creating files they make them compatible with other programs.

### Specials

A special highlight in MyChemise is the option of presenting any created drawings, structural images and texts in different ways; this can be done directly in the program itself. One of the options available allows you to put together different documents into a script in order to present a slide show. Another option exists that, for example, can arrange the fluctuating border structures of chemical depictions into an animation. Such animations can of course be integrated into a script. Morphing as a means of teaching chemistry/science has up until now been used very little. MyChemise offers you morphing as method of presentation.

### The morphing module

When morphing is carried out, two images are brought together. This involves allocating those areas of the images with each other that are to be transformed. Changes are made in steps and apply to both shape and colour. Intermediate steps are interpolated from the starting images, whereby the share of one image reduces in dimensions while the dimensions of the other image increase [8]. Using MyChemise, images can be morphed using affine or three-point mapping (i.e. division into triangular sections, affine mapping) and by dividing up into square areas. Four-point mapping (projective mapping) is mathematically solved using the unit squares method [8].

When illustrating chemical states, it is sometimes more useful to transform only specific areas into each other. MyChemise achieves this by automatically recognising only the bonding and atom areas in the structural images used as being areas to be morphed. As soon as only two drawings have been made, dynamic representations can then be quickly produced by calculating the intermediate steps. These allow movements (e.g. molecular vibrations) and sequences (e.g. reaction mechanisms) to be graphically simulated.

**Figure 10:**
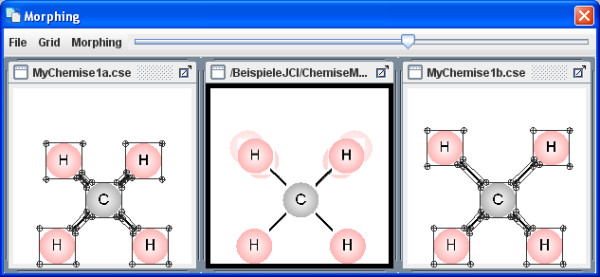
**Example Morph 1**. The morphing window shows an example of molecule vibrations (symmetrical stretching).

**Figure 11:**
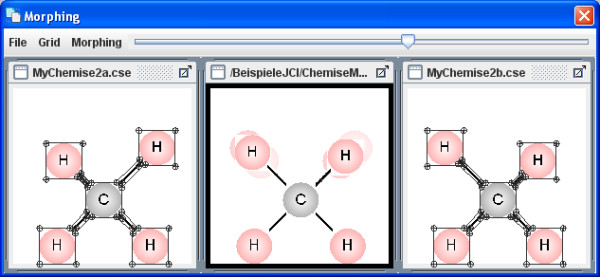
**Example Morph 2**. The morphing window shows an example of molecule vibrations (asymmetrical stretching).

**Figure 12:**
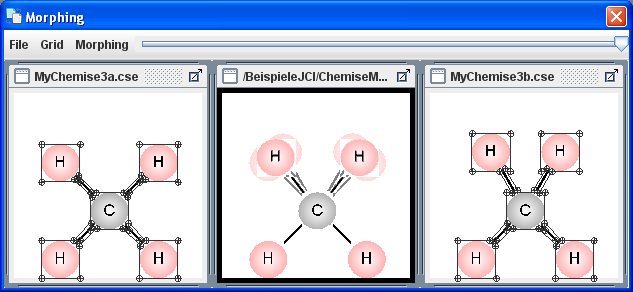
**Example Morph 3**. The morphing window shows an example of molecule vibrations (scissoring).

**Figure 13:**
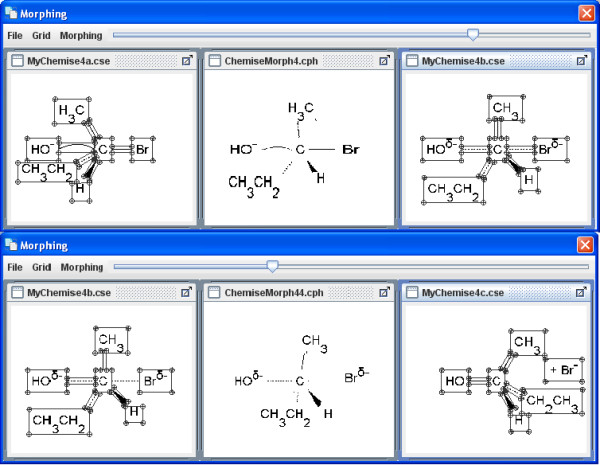
**A SN2-reaction as an example for a morphing sequence**. Reaction sequences can be visualized by combining two or more morphing steps.

The menu item Extras enables you to upload various, simple morphing examples in the on-line mode (Figures 10, 11, 12). The Morphing window then opens and they can be started from within the Morphing menu item. The process behaviour can be changed in the morphing set-up dialog box. Several morphing steps can be combined to a sequence (Figure 13).

## Conclusions

MyChemise is a new 2D drawing program that places special importance on simple operation and versatile ways of creating structural images. Continual advancements in processors have led to increasingly faster desktop PCs. Greater amounts of RAM have also enabled the inclusion of methods for displaying dynamic processes in such programs, in this case the morphing module.

An enhancement for MyChemise is currently being worked on, which will, amongst other things, be able to export SMILES strings [9] that can be used for structure searching in databases.

The continuation of MyChemise as an open source project has been planned for a later date.

## Competing interests

The author declares that they have no competing interests.

## Supplementary Material

Additional file 1 **Description**. The software description of MyChemise describes and presents the menu items. Well-known commands from standard-software (save, open etc.) or self-explanatory commands are not included. Click here for file

Additional file 2 **mychemise**. It contains two files (MyChemise.html and ChemJar.jar). It can be downloaded and installed for running MyChemise in the off-line mode, too. Click here for file
