# Supplementary material for: MyChemise: A 2D drawing program that uses morphing for visualisation purposes
Source: J Cheminform. 2011 Dec 12;3:53. doi: 10.1186/1758-2946-3-53 (PMC3264509; doi:10.1186/1758-2946-3-53)
Supplement: Additional file 1 — Description. The software description of MyChemise describes and presents the menu items. Well-known commands from standard-software (save, open etc.) or self-explanatory commands are not included. [file 1758-2946-3-53-S1.PDF]

MyChemise

My Chemical Structure Editor

Software description

by Dr. Jörg-Hubertus Wilhelm, May 2011

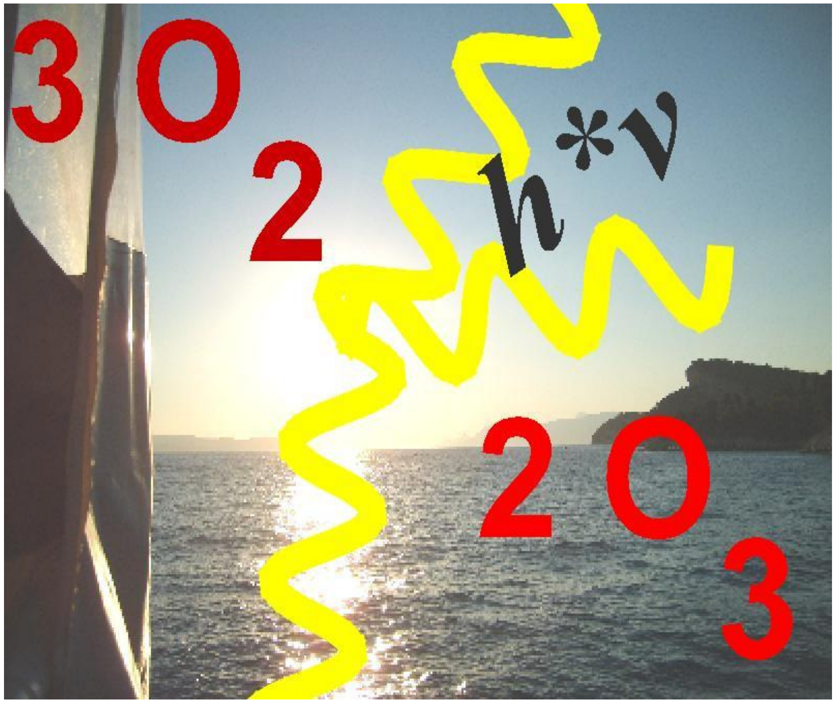

Contents

Introduction

1 The menu

1.1 The file menu item
1.1.1 The set-up sketch area window

1.2 The edit menu item

1.3 The paste menu item

1.4 The atom menu item

1.5 The bond menu item

1.6 The bond-cross menu item

1.7 The view menu item

1.8 The presentation menu item
1.8.1 The scripting toolbar
1.8.2 The windows scripting dialog
1.8.3 The animation dialog window
1.8.4 Morphing window
1.8.4.1 Set-up (morphing) dialog window
1.8.4.2 Sequence dialog window
1.8.4.3 Allocation dialog window

2 Procedure for writing a script

3 Making an animation

4 Morphing

4.1 Making a morphing sequence

5 Note for Linux users

Introduction

MyChemise (my-chemical-structure-editor) is a 2D chemical structure drawing program that can present work in different ways.
MyChemise is a Java-Applet. It runs as an application in a browser-window. MyChemise needs an up-to-date Java plug-in.
If the plug-in has not already been installed it will normally install automatically into the Microsoft operating system being used when MyChemise starts for the first time. If this doesn't happen (e.g. under Linux) then the plug-in has to be installed manually (first try it with http://java.com/en/download/installed.jsp) There you can normally find a link for the plug-in.
If not, browse for this Java plug-in).

Normally, for security reasons an applet is embedded in a 'sandbox' .
However, in MyChemise, you can use the clipboard to copy and paste. Files can also be saved on a computer's own hard disc.
For this reason, MyChemise is a signed applet with extended access rights.
Consequently, you will get a system-dependent security warning when MyChemise starts up.

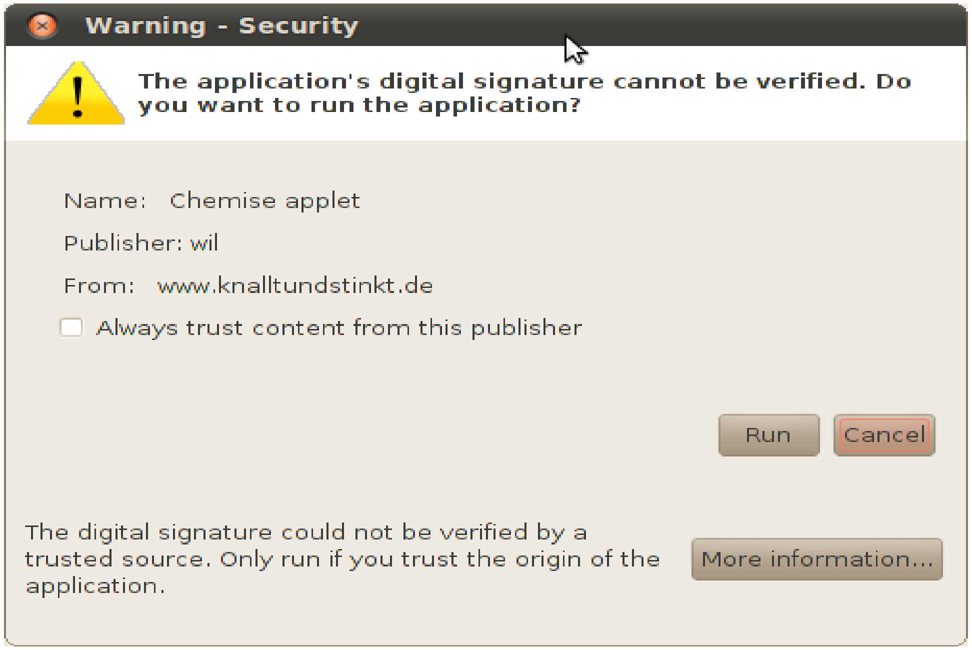

If you want to use MyChemise you have to agree to it

The following text describes and presents the menu items in MyChemise. Well-known commands from standard-software (save, open etc.) or self-explanatory commands are not described.

1 The menu

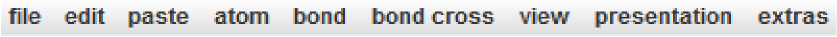

When the mouse pointer is moved over each item the corresponding toolbar appears.
Click on an item in the toolbar to fix it.

1.1 The file menu item

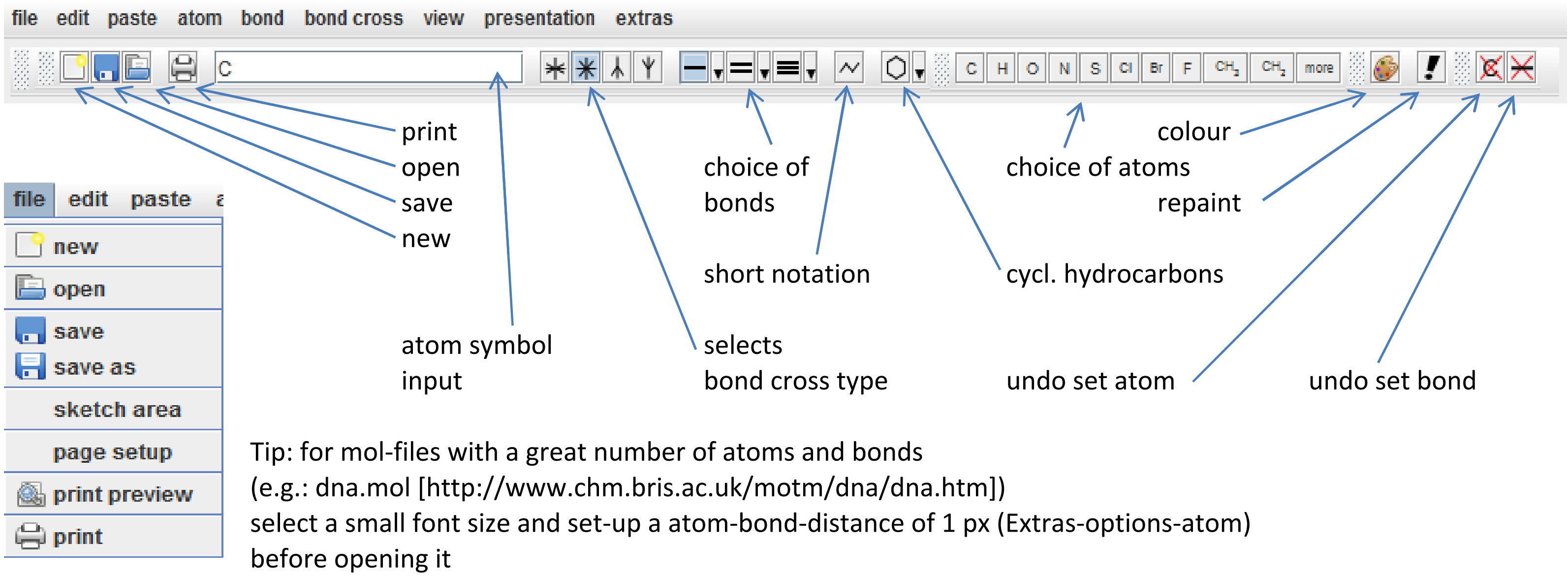

Tip: for mol-files with a great number of atoms and bonds
(e.g.: dna.mol [http://www.chm.bris.ac.uk/motm/dna/dna.htm])
select a small font size and set-up a atom-bond-distance of 1 px (Extras-options-atom)
before opening it

1.1.1 The set-up sketch area window

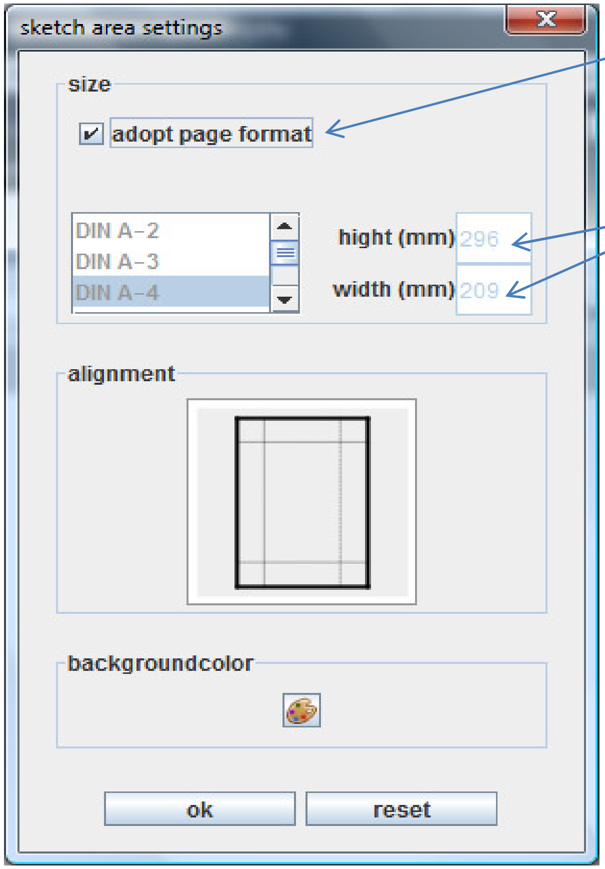

The adopt page format (file --> page set-up) is set by
by default. The standard setting is a DIN A-4 page in portrait format

Can be changed

Tip: MyChemise is a drawing program. The dimensions of the sketch area directly effect the computer's speed.
Therefore, select the smallest, necessary dimensions that you can use.

1.2 The edit menu item

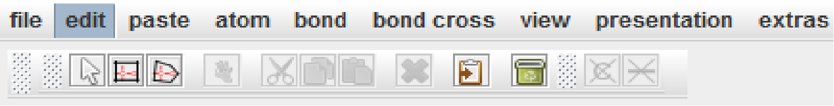

Note: in the edit menu it is impossible to set a new atom or a bond using a mouse click. To do this, you must first change back to the file, atom or bond menu.

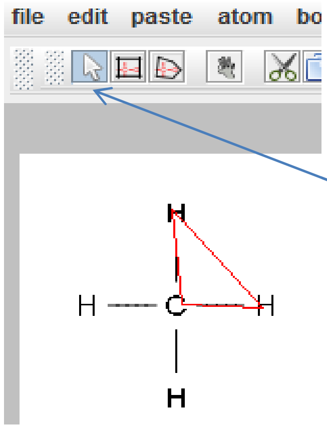

to select specific atoms and bonds: click on each atom/bond

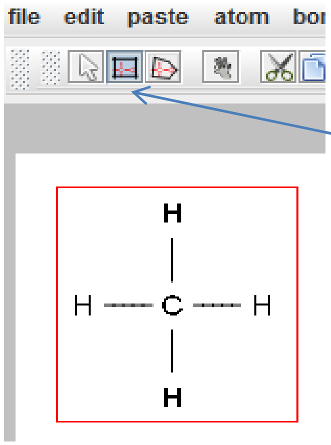

rectangular selection frame: press mouse and drag

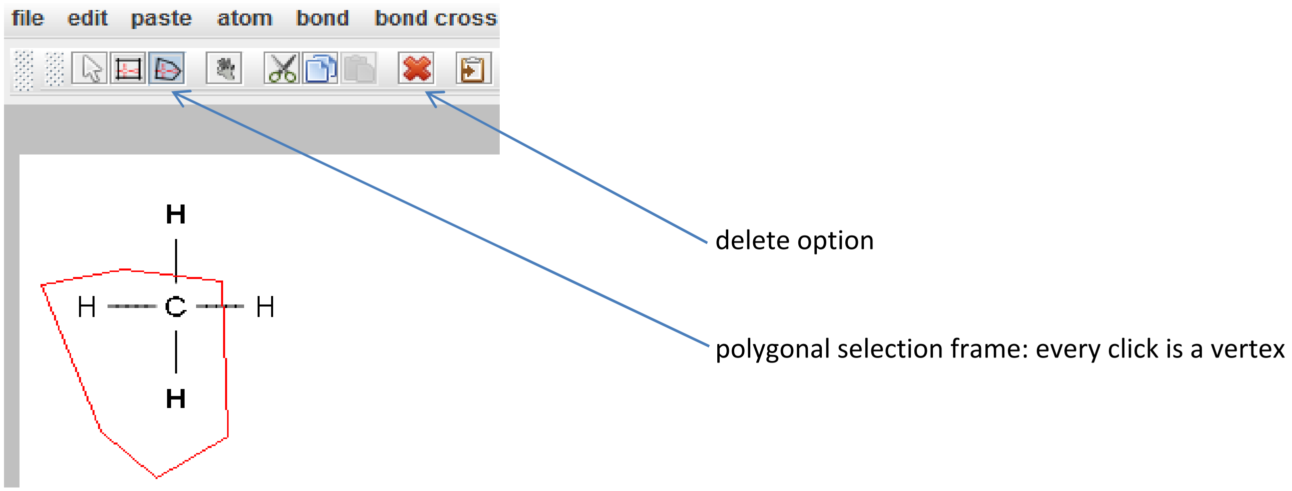
file edit paste atom bond bond cross
H
H C H
H
delete option
polygonal selection frame: every click is a vertex

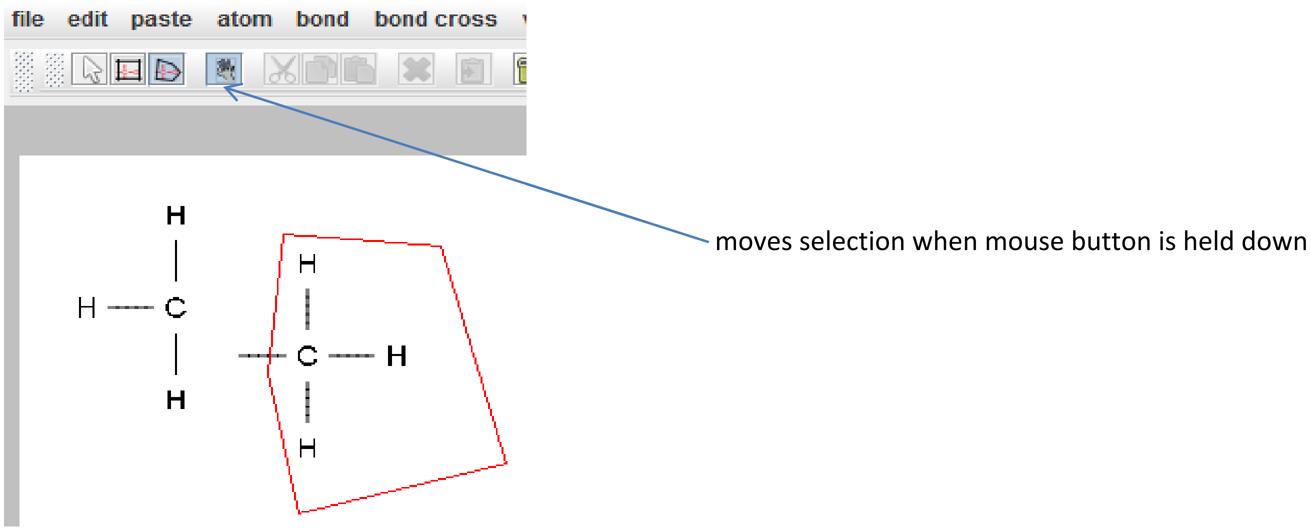
file edit paste atom bond bond cross
H
H C
H
H
C H
H
moves selection when mouse button is held down

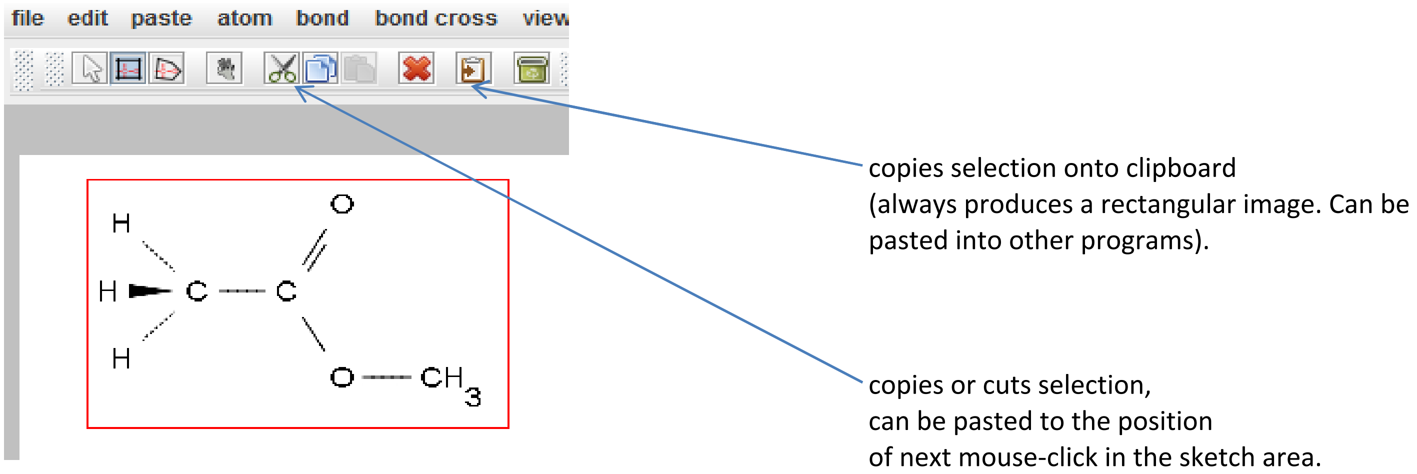

1.3 The paste menu item

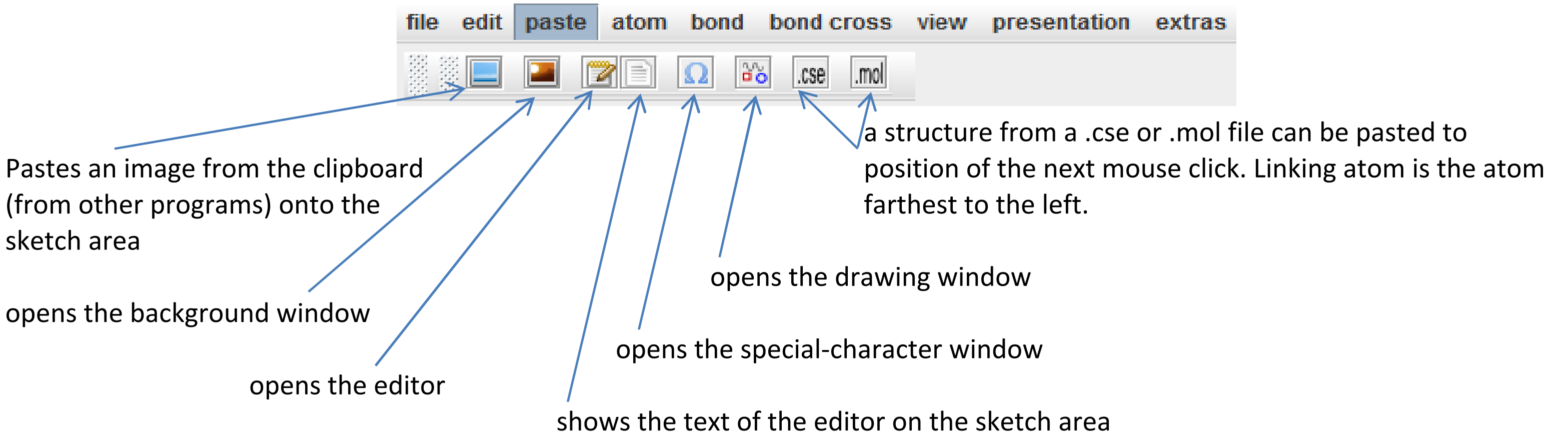

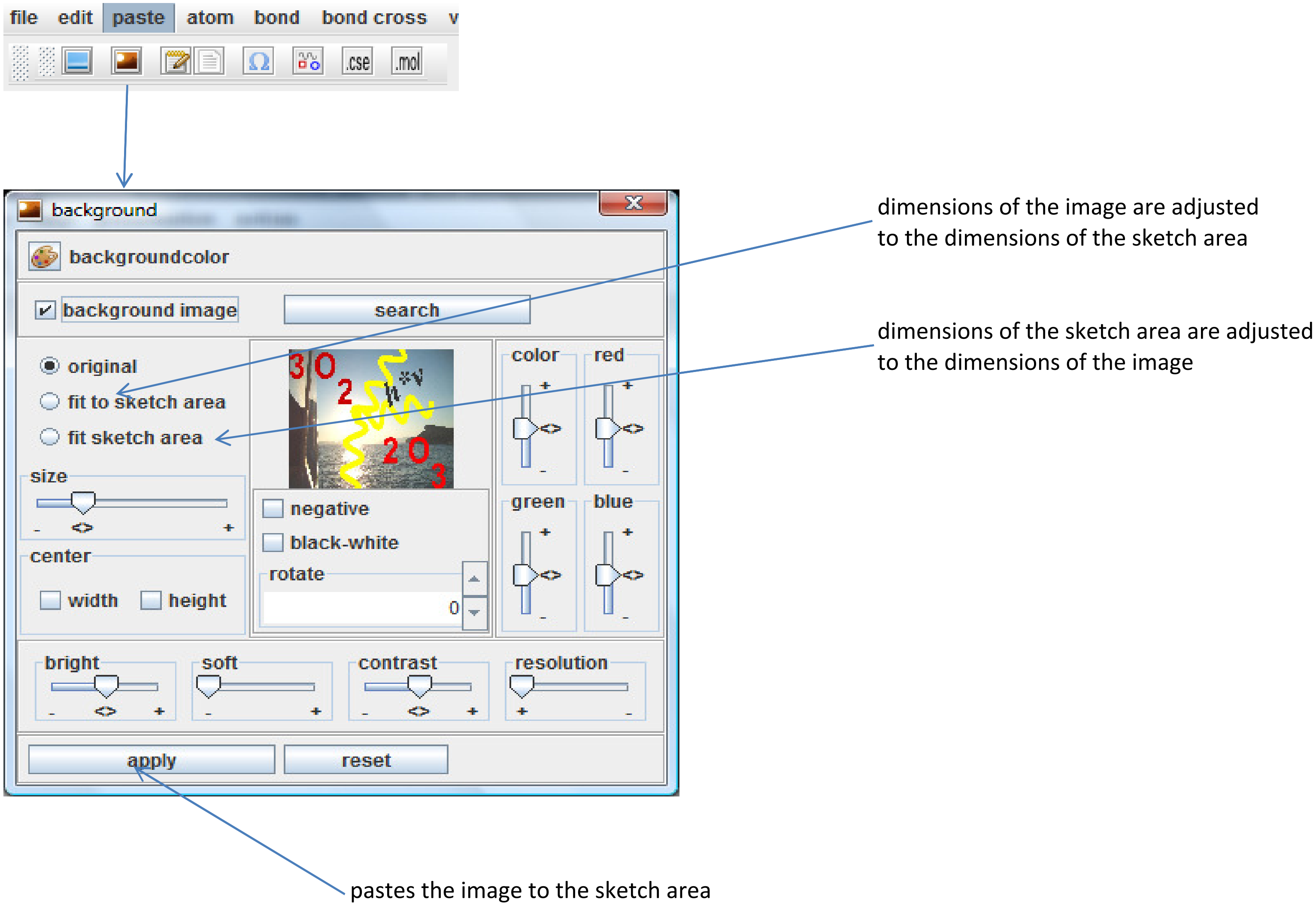
file edit paste atom bond bond cross v
.cse .mol
background
backgroundcolor
background image
search
original
fit to sketch area
fit sketch area
size
center
width
height
negative
black-white
rotate
0
color
red
green
blue
bright
soft
contrast
resolution
apply
reset
dimensions of the image are adjusted to the dimensions of the sketch area
dimensions of the sketch area are adjusted to the dimensions of the image
pastes the image to the sketch area

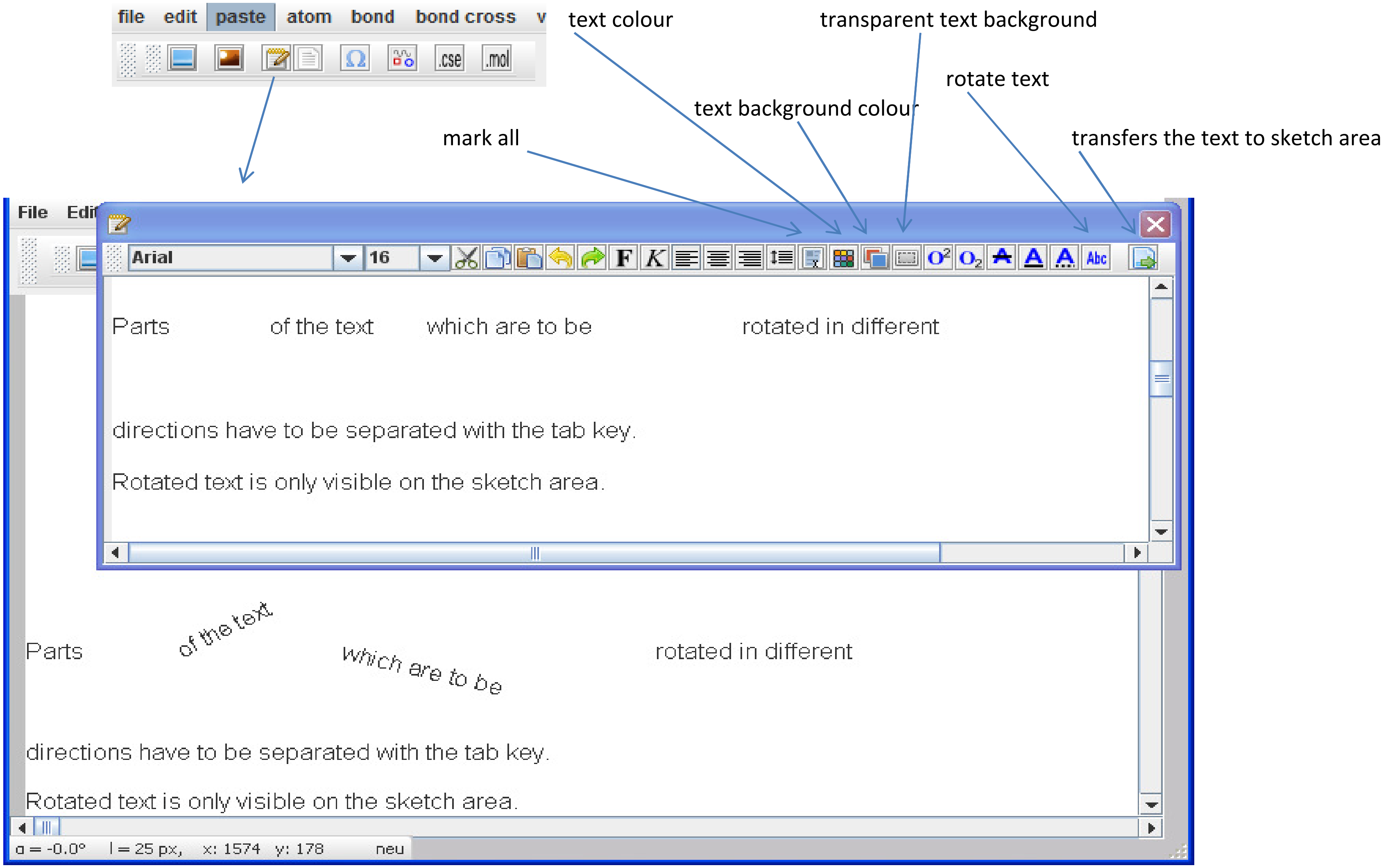
file edit paste atom bond bond cross v
text colour
transparent text background
rotate text
text background colour
mark all
transfers the text to sketch area
File Edit
Arial
16
Parts of the text which are to be rotated in different
directions have to be separated with the tab key.
Rotated text is only visible on the sketch area.
Parts of the text which are to be rotated in different
directions have to be separated with the tab key.
Rotated text is only visible on the sketch area.
α = -0.0° l = 25 px, x: 1574 y: 178 neu

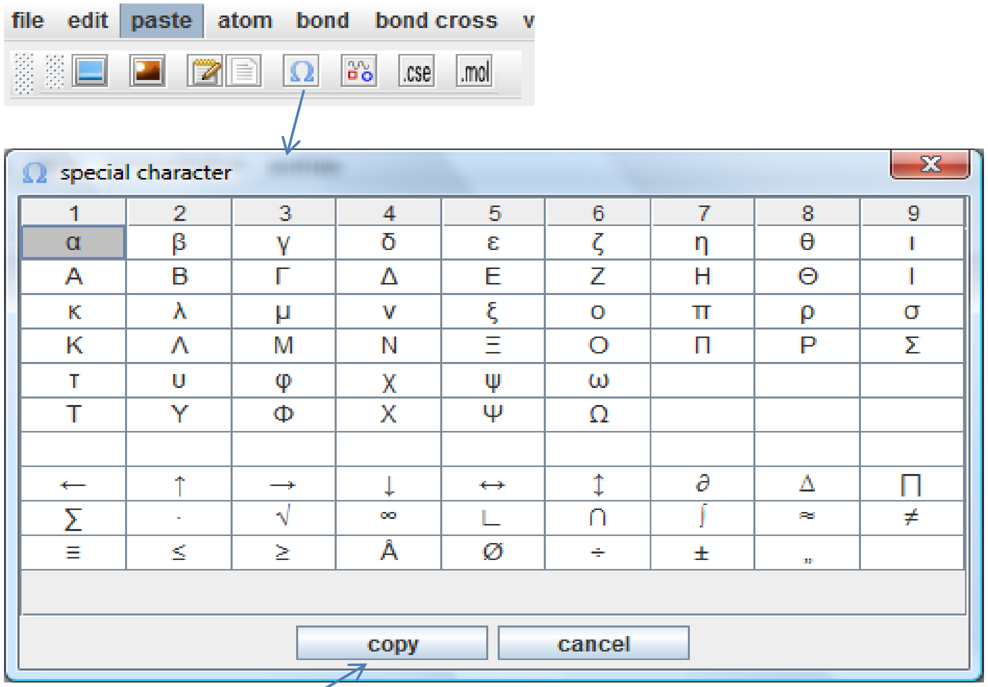

copy to the clipboard
(you can paste it into the editor or into the atom symbol input -->

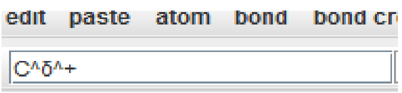

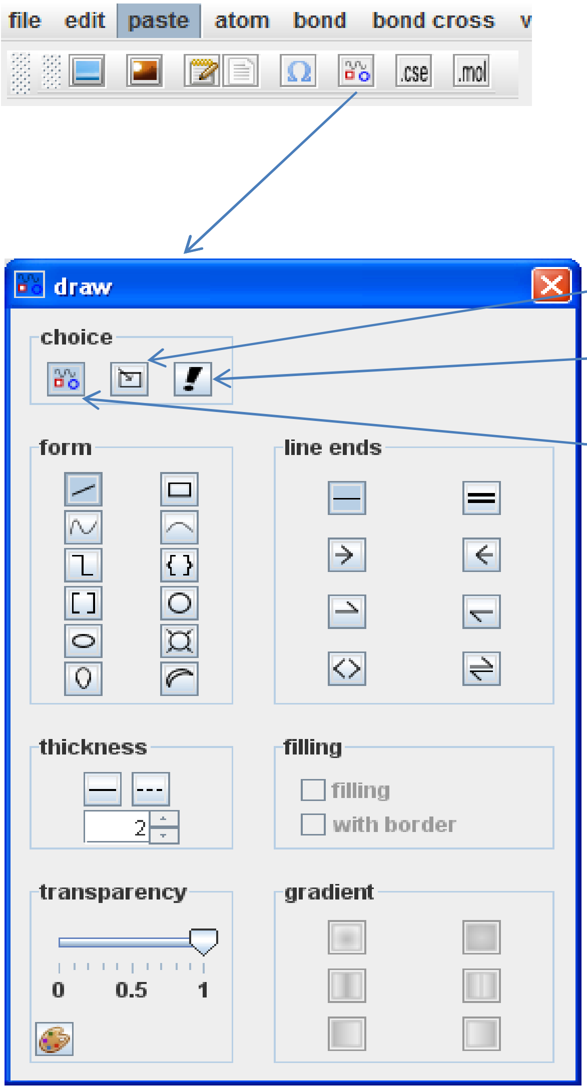

select a form with a mouse click for changing the size, for moving etc.

repaint (sketch area will be refreshed)

you can draw by keeping the mouse button depressed while on the sketch area

1.4 The atom menu item

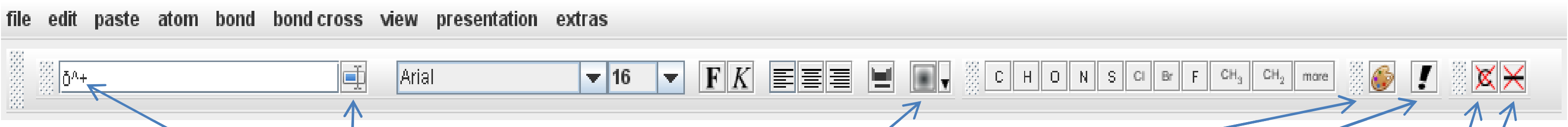

text box: you can e. g. place numbers close to atom symbols (you have to enter your input here and then you have to click the sketch-area)

atomic shells

text colour

repair (sketch area will be refreshed)

undo set atom

undo set bonds

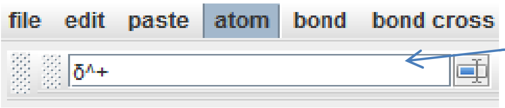

input for atom symbols or short text
e.g.: (superscript with ^) O^2^ +
(subscript with _) O_2
(write in left text direction with <) He<^4
(marker sign for < set using #) ^4#He<_2

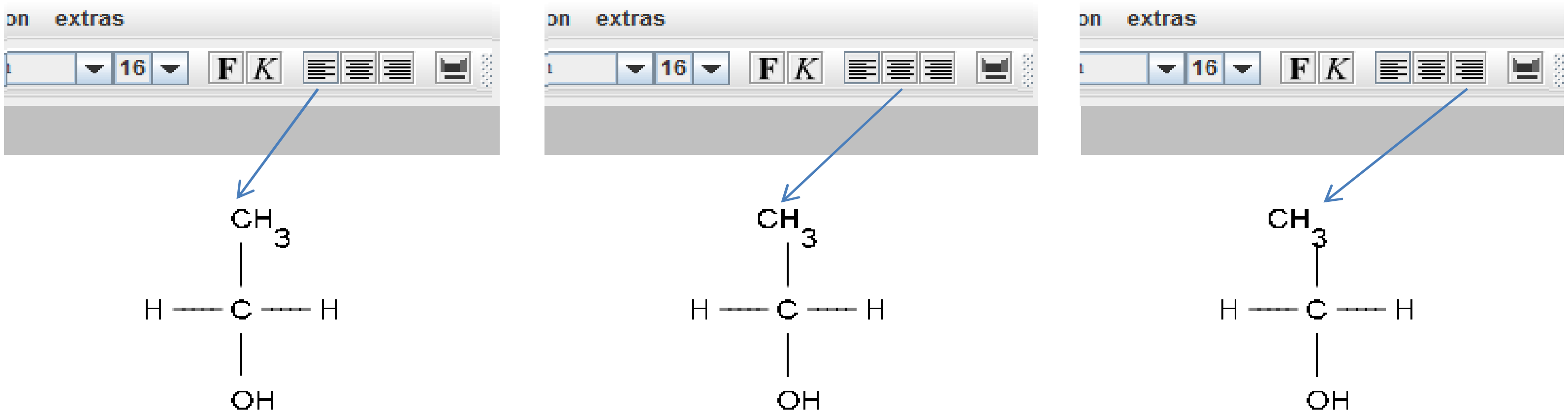

Text alignment can only be adjusted before setting an atom.
Therefore, subsequent changes require that you first delete the atom, realign and then re-enter the text.

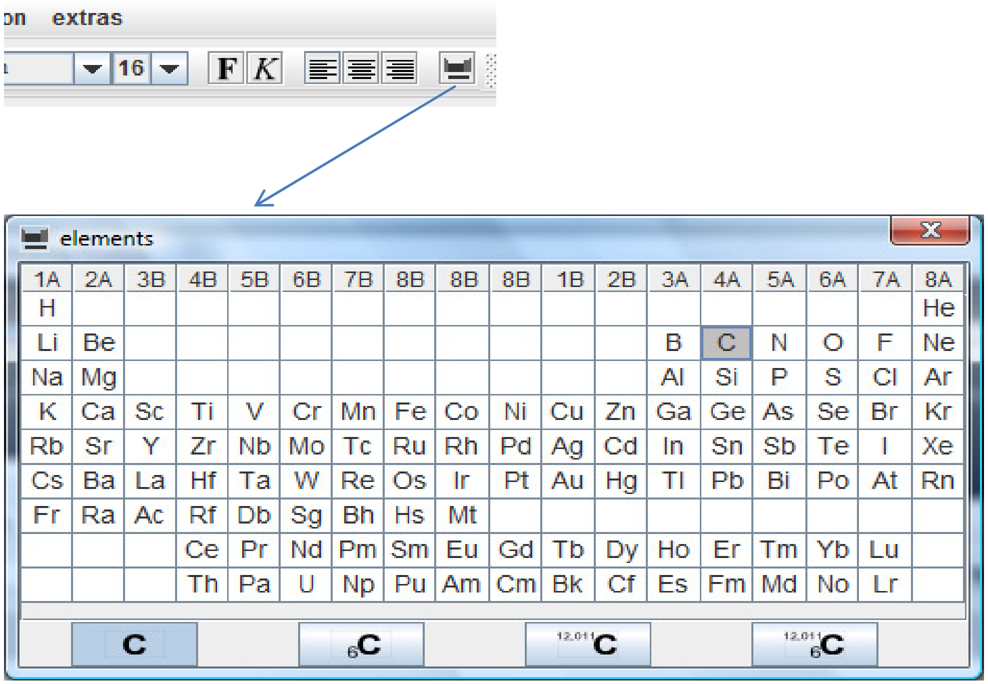
extras
16
F K
elements
1A 2A 3B 4B 5B 6B 7B 8B 8B 8B 1B 2B 3A 4A 5A 6A 7A 8A
H He
Li Be B C N O F Ne
Na Mg Al Si P S Cl Ar
K Ca Sc Ti V Cr Mn Fe Co Ni Cu Zn Ga Ge As Se Br Kr
Rb Sr Y Zr Nb Mo Tc Ru Rh Pd Ag Cd In Sn Sb Te I Xe
Cs Ba La Hf Ta W Re Os Ir Pt Au Hg Tl Pb Bi Po At Rn
Fr Ra Ac Rf Db Sg Bh Hs Mt
Ce Pr Nd Pm Sm Eu Gd Tb Dy Ho Er Tm Yb Lu
Th Pa U Np Pu Am Cm Bk Cf Es Fm Md No Lr
C
6C
12,011C
12,011 6C

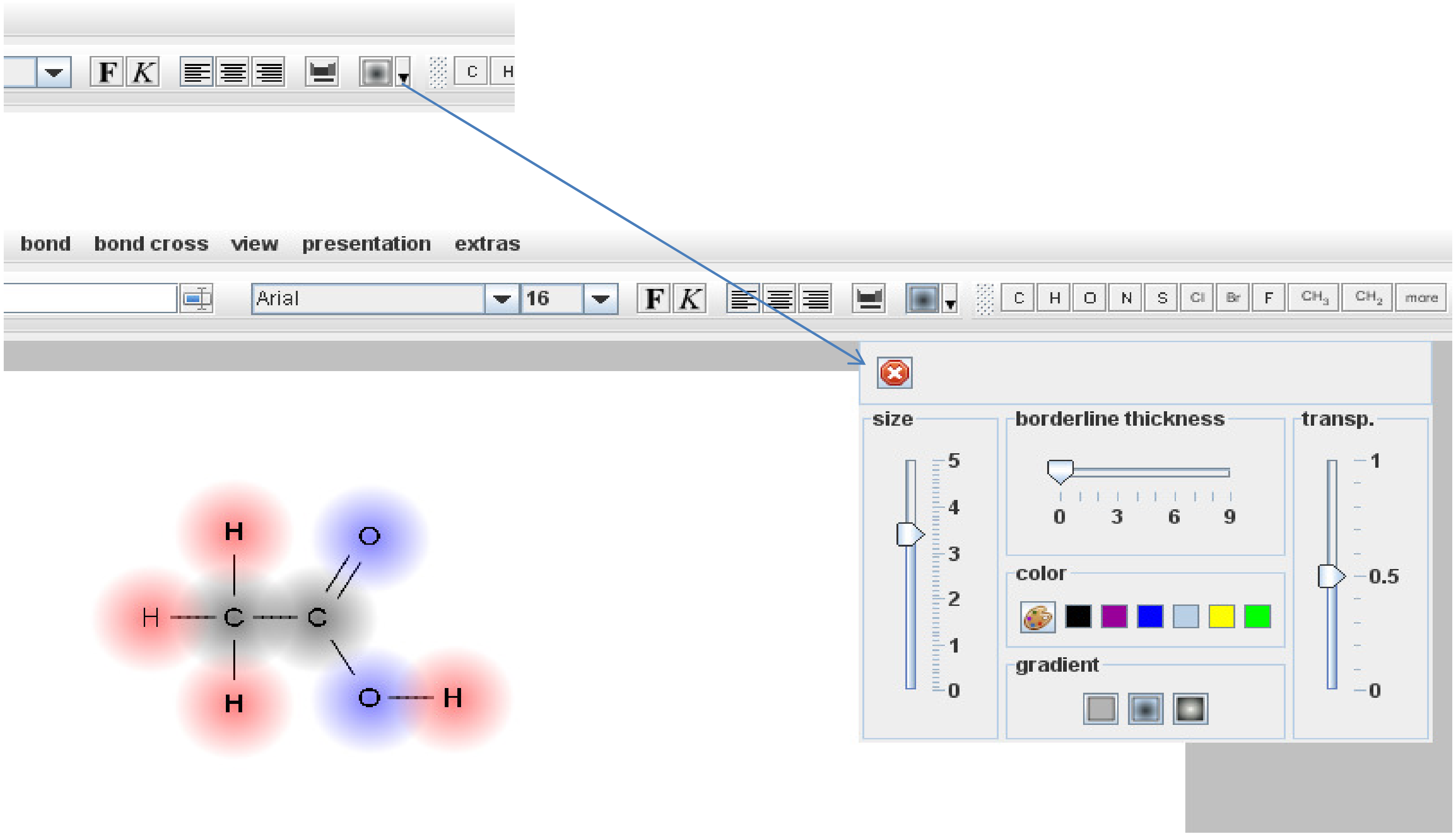
bond
bond cross
view
presentation
extras
Arial
16
C
H
O
N
S
Cl
Br
F
CH3
CH2
more
size
borderline thickness
transp.
color
gradient
5
4
3
2
1
0
0
3
6
9
1
0.5
0
H
H
H
O
C
C
O
H

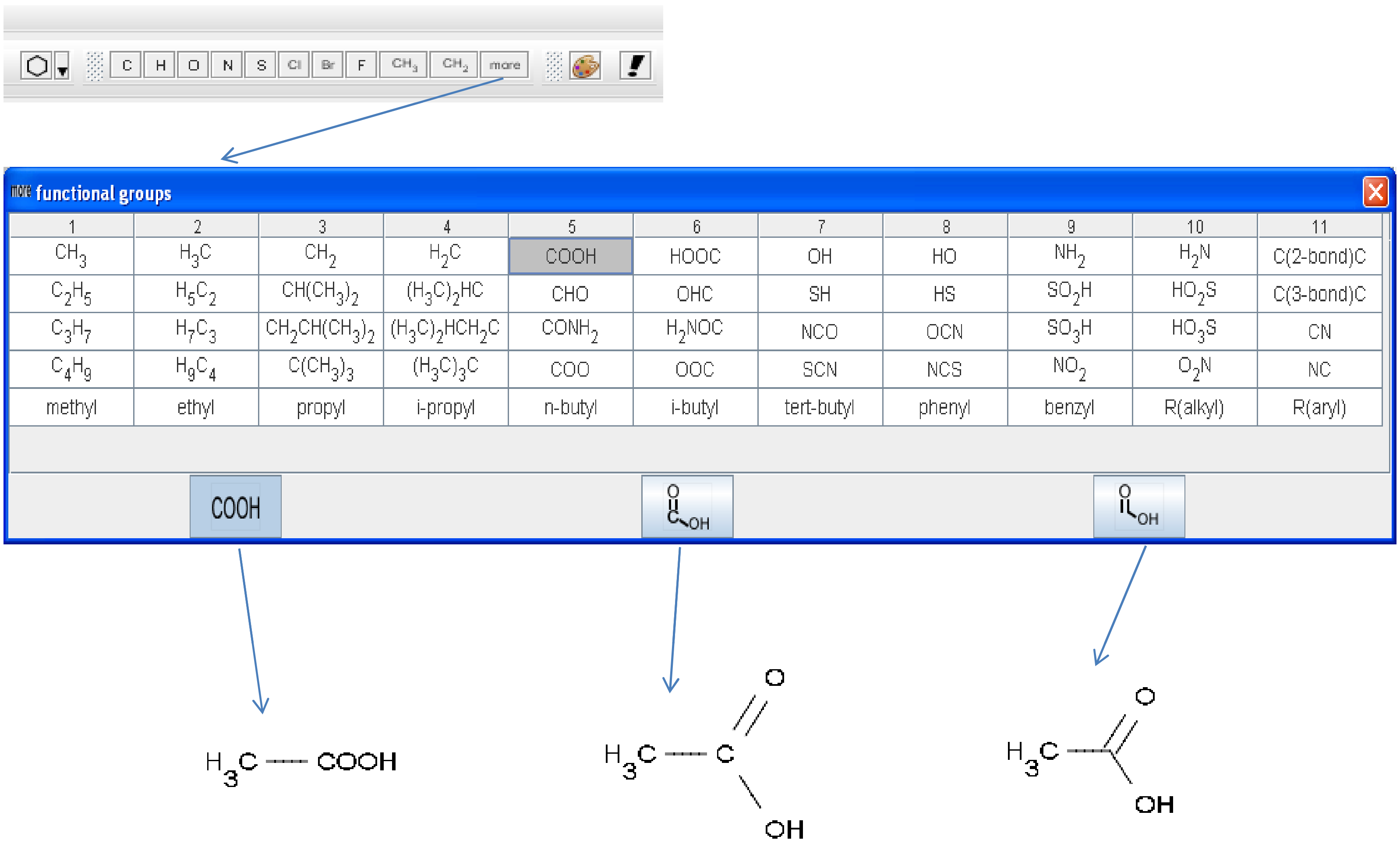
C H O N S Cl Br F CH3 CH2 more
functional groups
1 2 3 4 5 6 7 8 9 10 11
CH3 H3C CH2 H2C COOH HOOC OH HO NH2 H2N C(2-bond)C
C2H5 H5C2 CH(CH3)2 (H3C)2HC CHO OHC SH HS SO2H HO2S C(3-bond)C
C3H7 H7C3 CH2CH(CH3)2 (H3C)2HCH2C CONH2 H2NOC NCO OCN SO3H HO3S CN
C4H9 H9C4 C(CH3)3 (H3C)3C COO OOC SCN NCS NO2 O2N NC
methyl ethyl propyl i-propyl n-butyl i-butyl tert-butyl phenyl benzyl R(alkyl) R(aryl)
COOH
H3C — COOH

1.5 The bond menu item

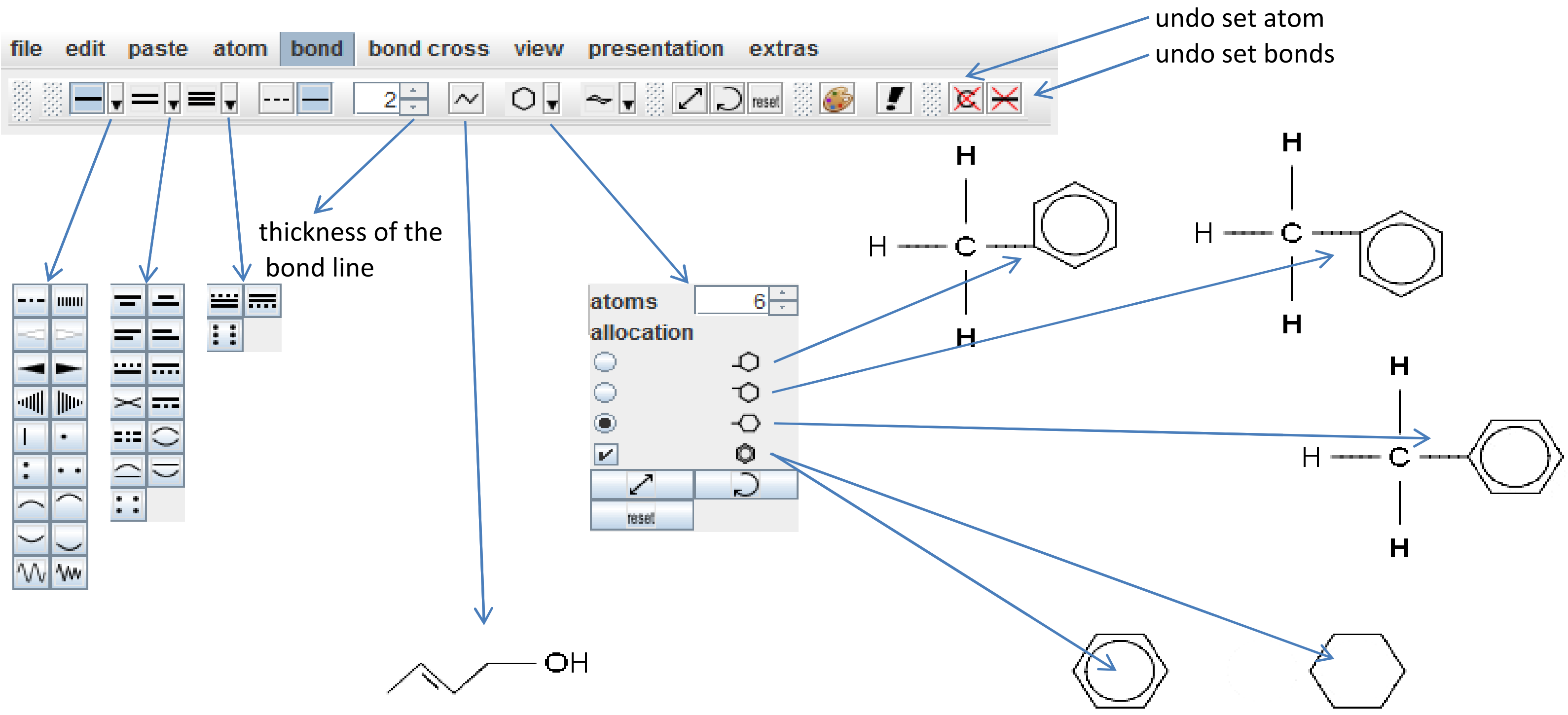

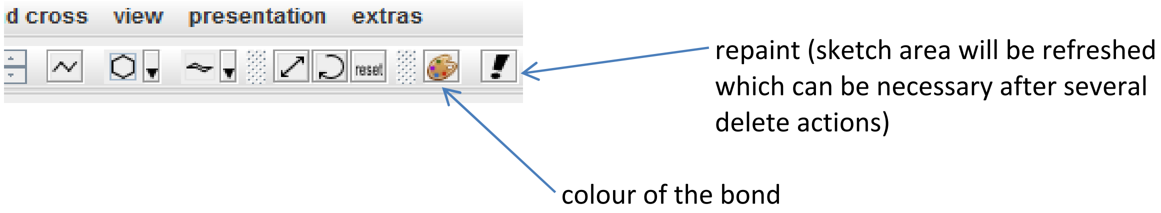

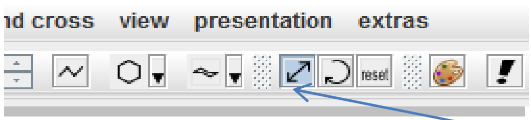

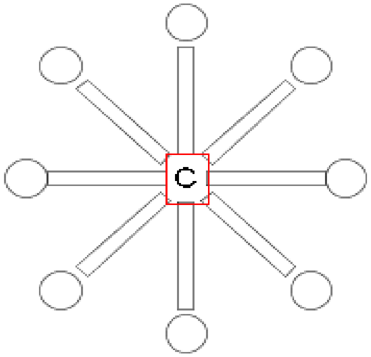

turns or drags the bond cross
for changing the angle or
bond length

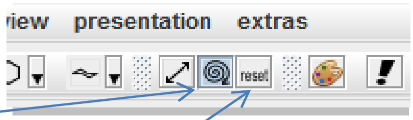

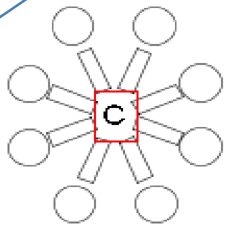

resets the bond length to standard value (extras - options)
and turns the bond cross to 0°

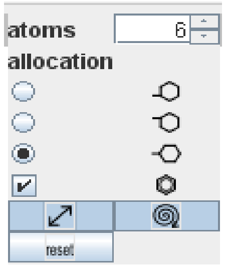

first press here then turn resp. drag

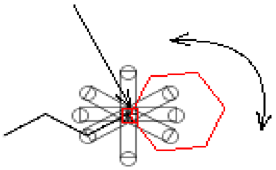

1.6 The bond-cross menu item

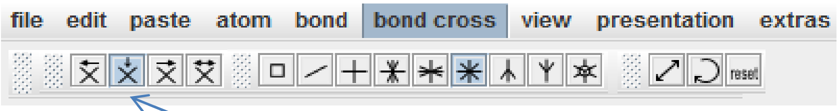

Setting the position of the bond cross:

shifted to the right

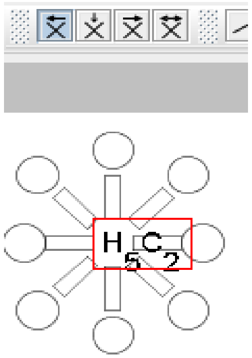

centralised

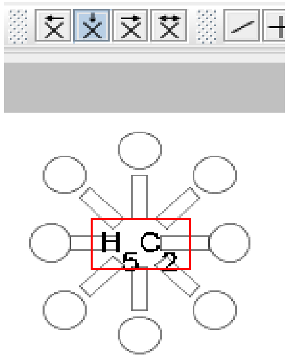

shifted to the left

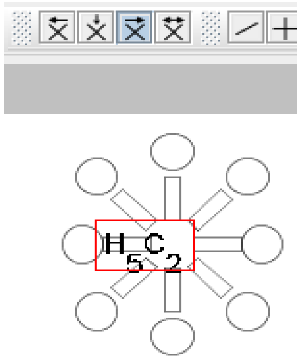

stretched

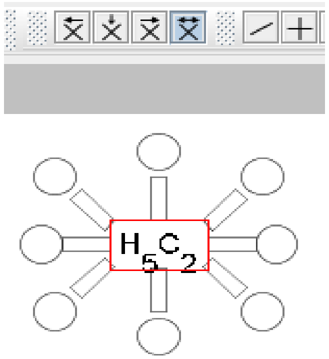

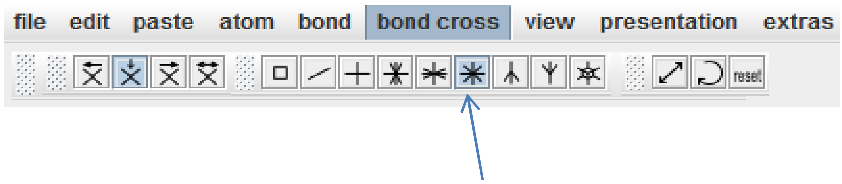

Selects type of bond cross:

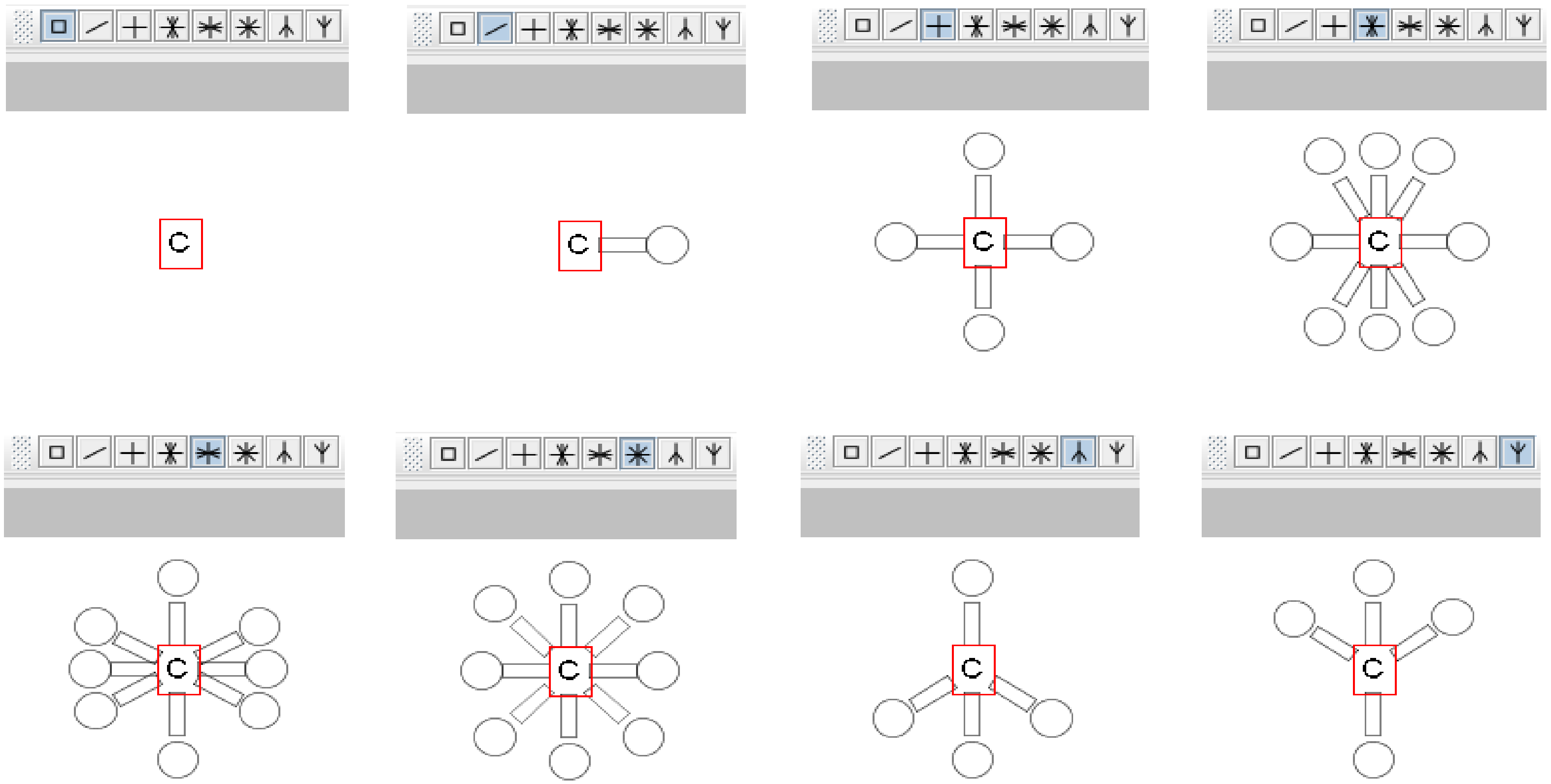

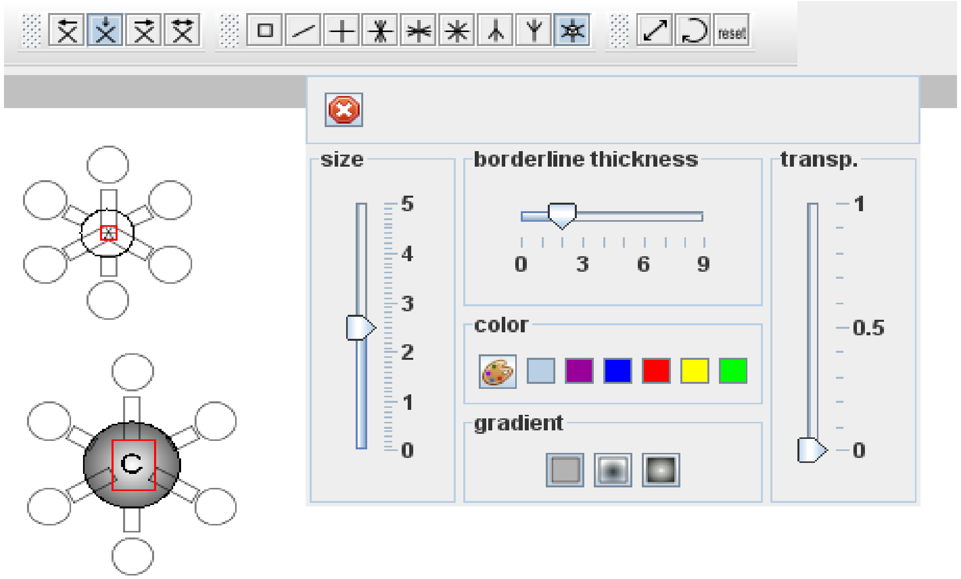

for Newman projections you can change the appearance of the circle using the atomic shell panel

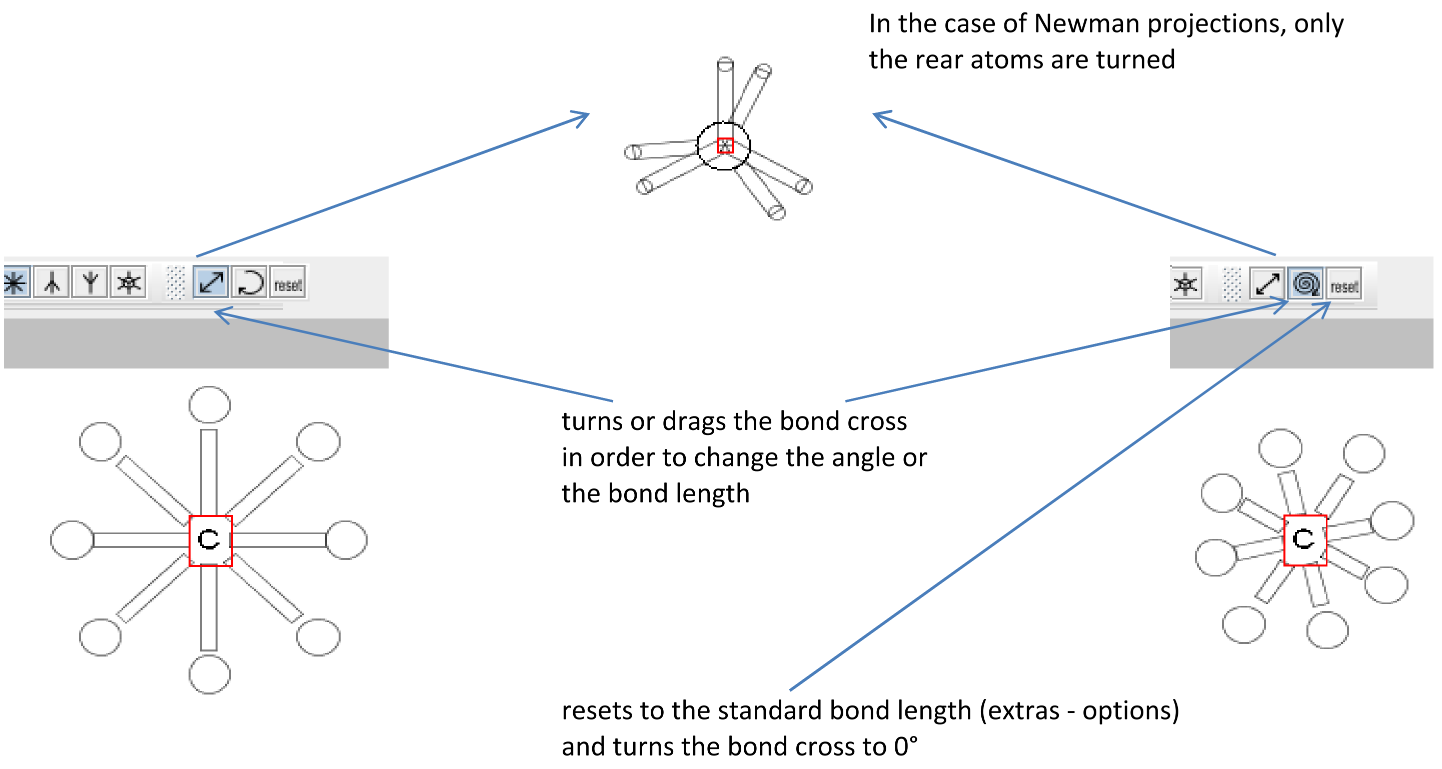
In the case of Newman projections, only the rear atoms are turned
reset
reset
C
C
turns or drags the bond cross in order to change the angle or the bond length
resets to the standard bond length (extras - options) and turns the bond cross to 0°

1.7 The view menu item

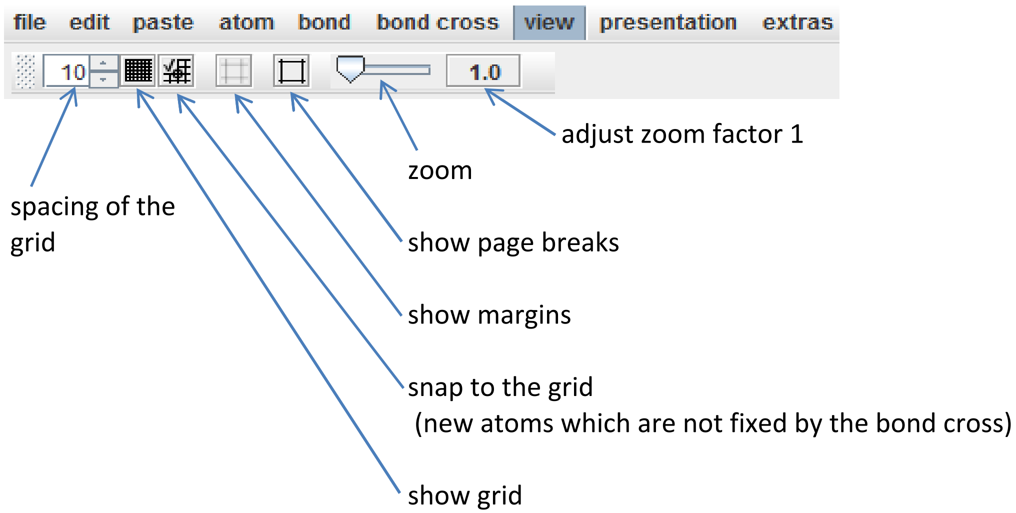

1.8 The presentation menu item

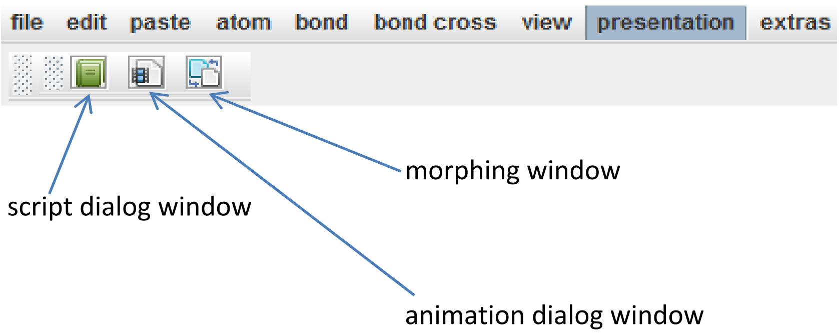

1.8.1 The scripting toolbar

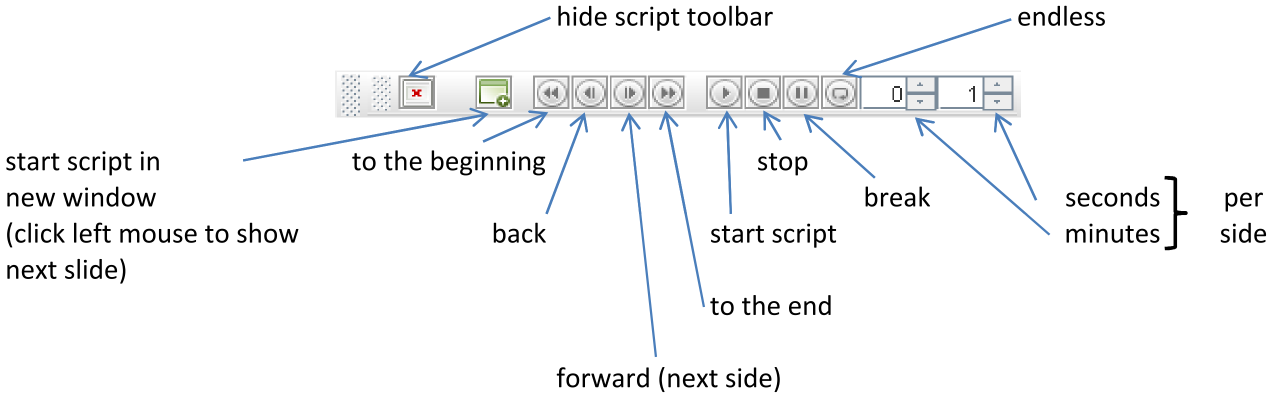

1.8.2 The windows scripting dialog

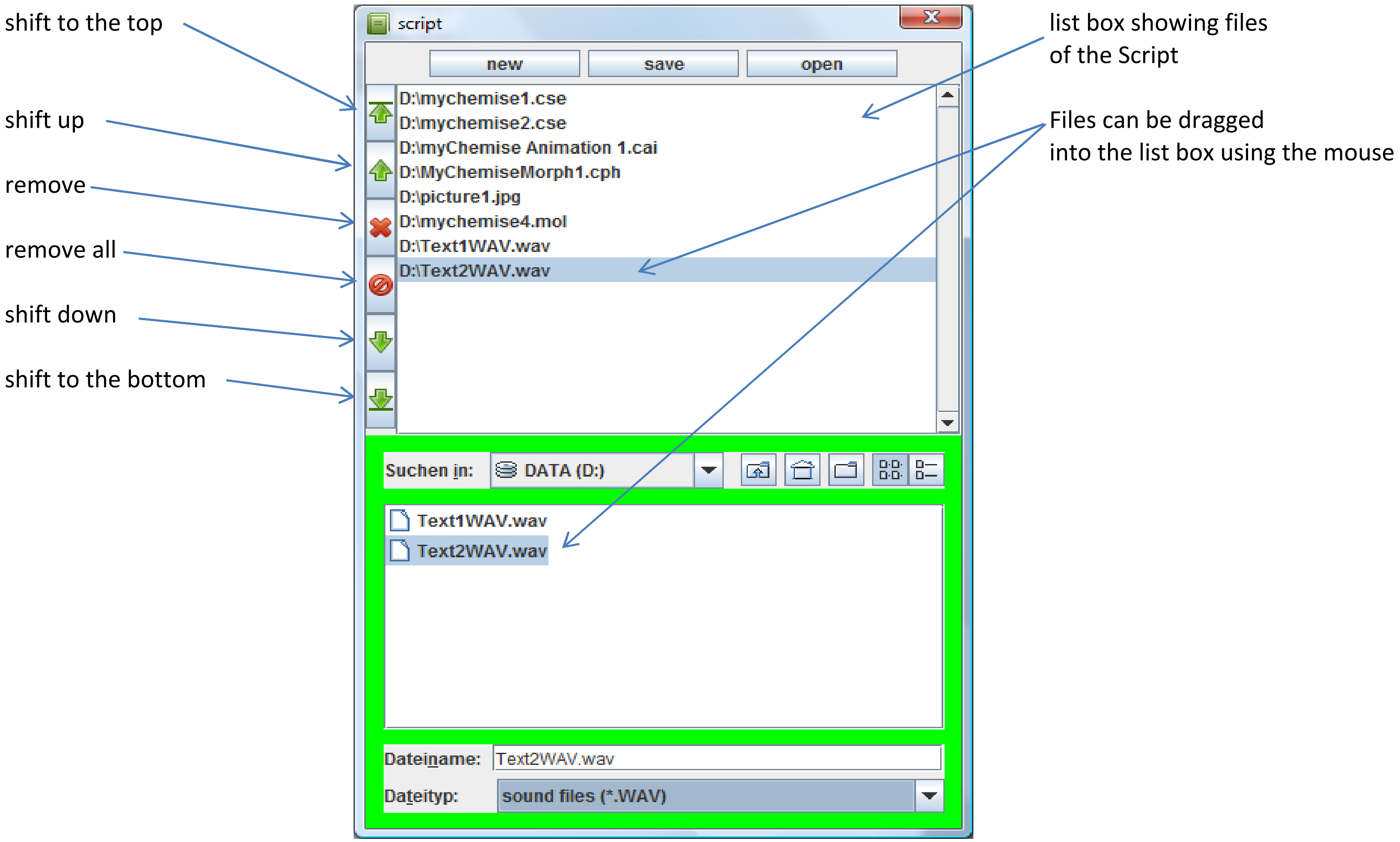

1.8.3 The animation dialog window

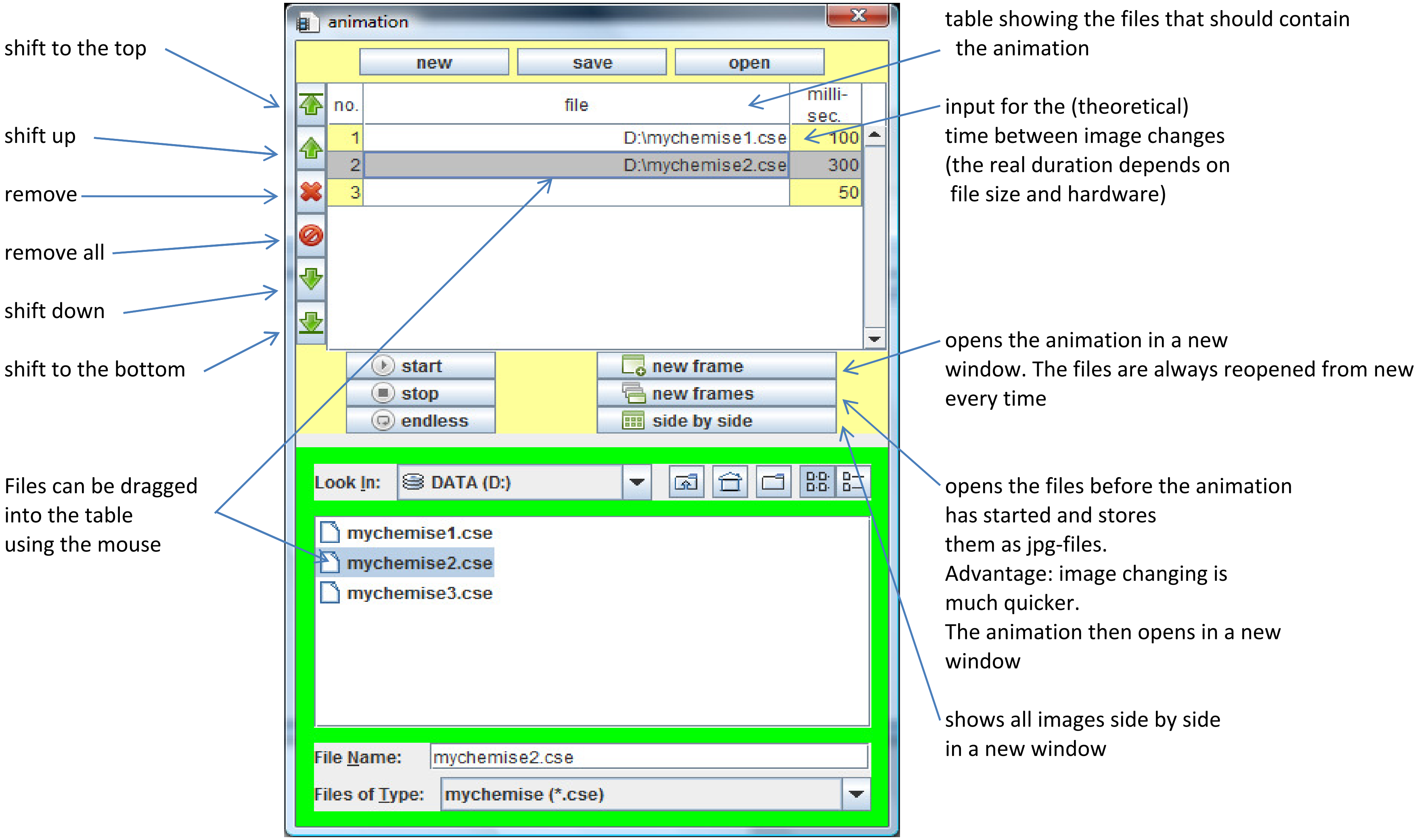

1.8.4 Morphing window

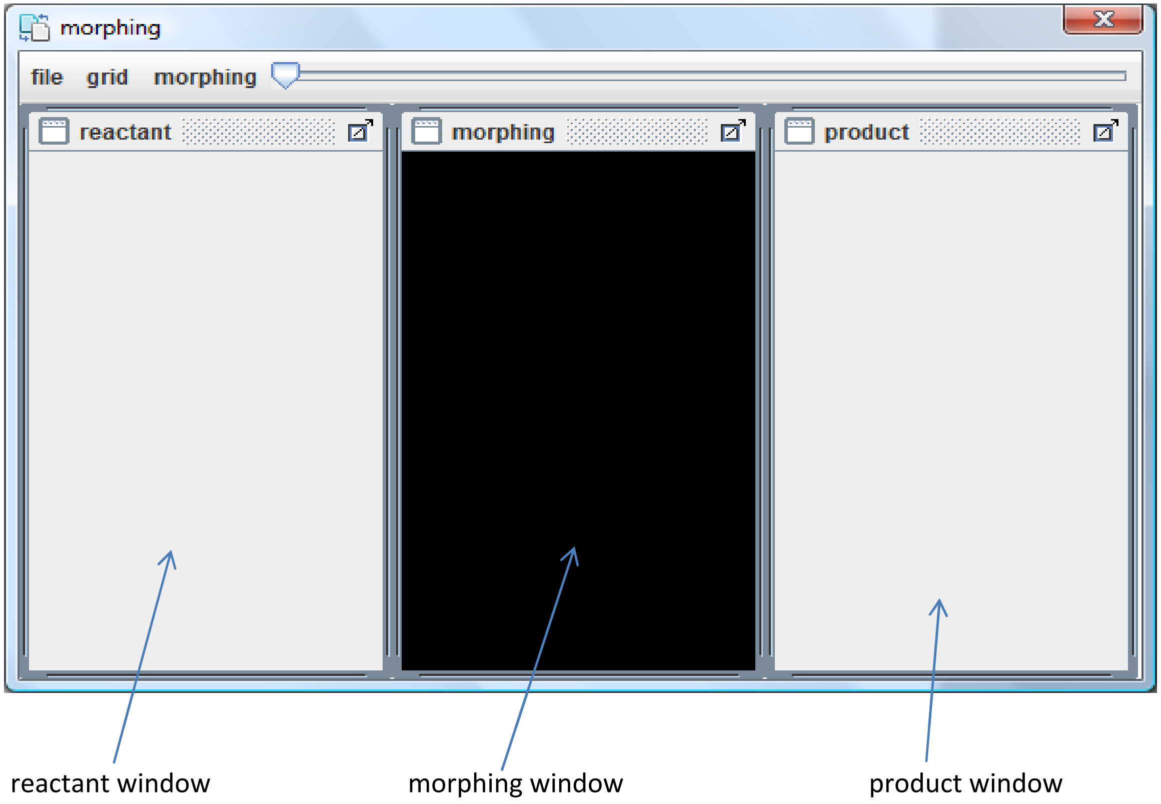

1.8.4.1 Set-up (morphing) dialog window

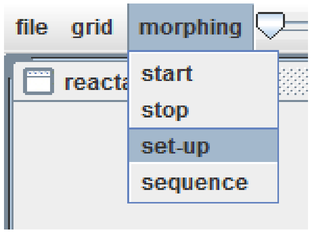

number of intermediate steps calculated

input for the (theoretical) time between image changes (the real duration depends on file size and hardware)

choice of grids for defining the morphing areas

number of rows and columns for 3 and 4 point affine mappings

Set the size - this is for freely placeable rectangles

behaviour of the morphing areas

behaviour of the background (non-morphing areas)

process behaviour

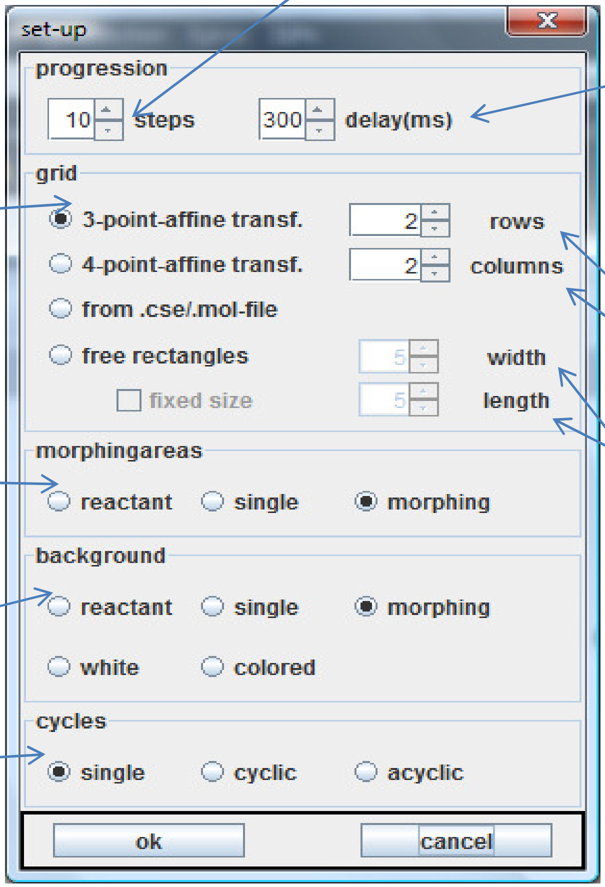

1.8.4.2 Sequence dialog window

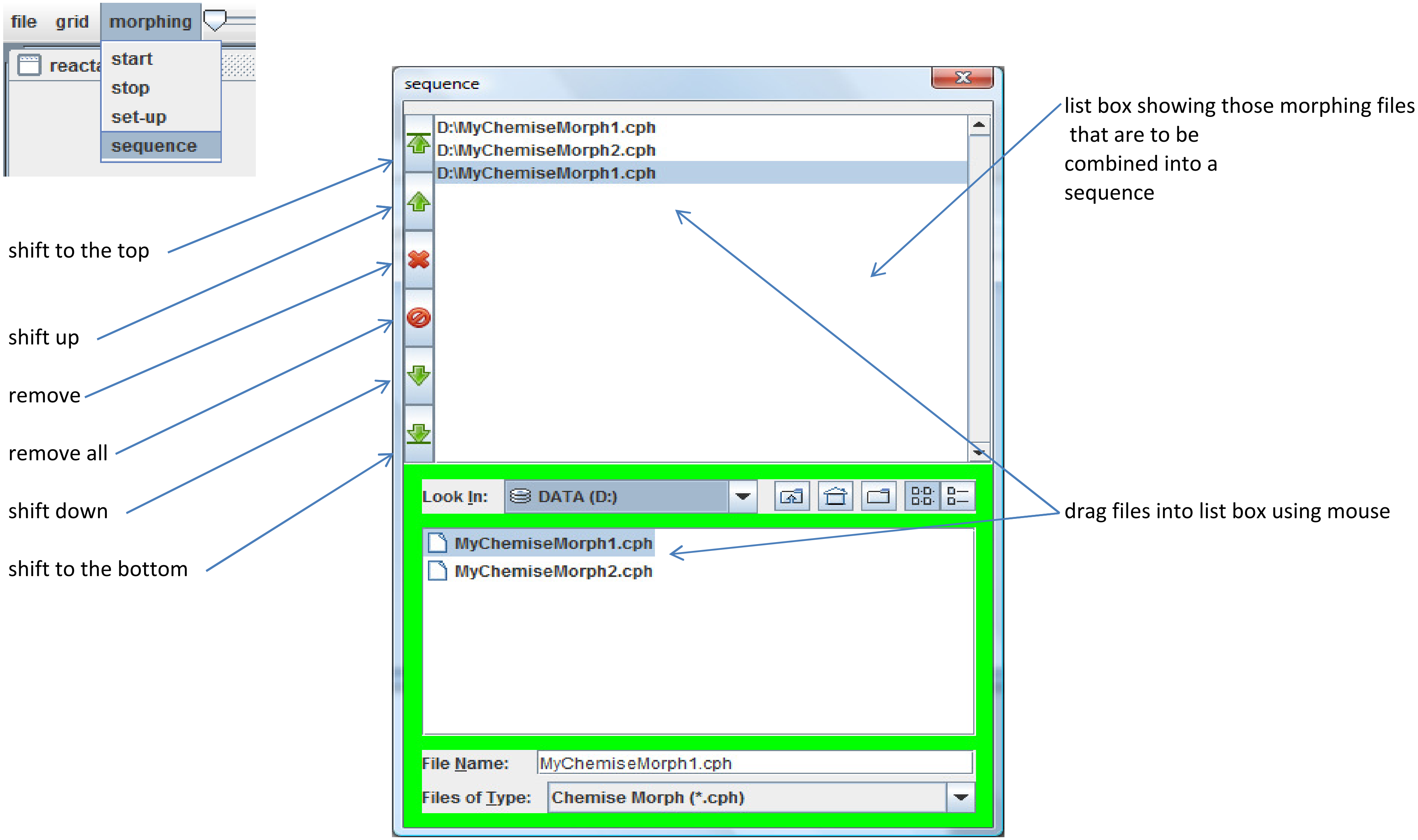

1.8.4.3 Allocation dialog window

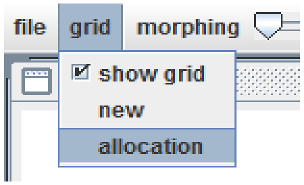

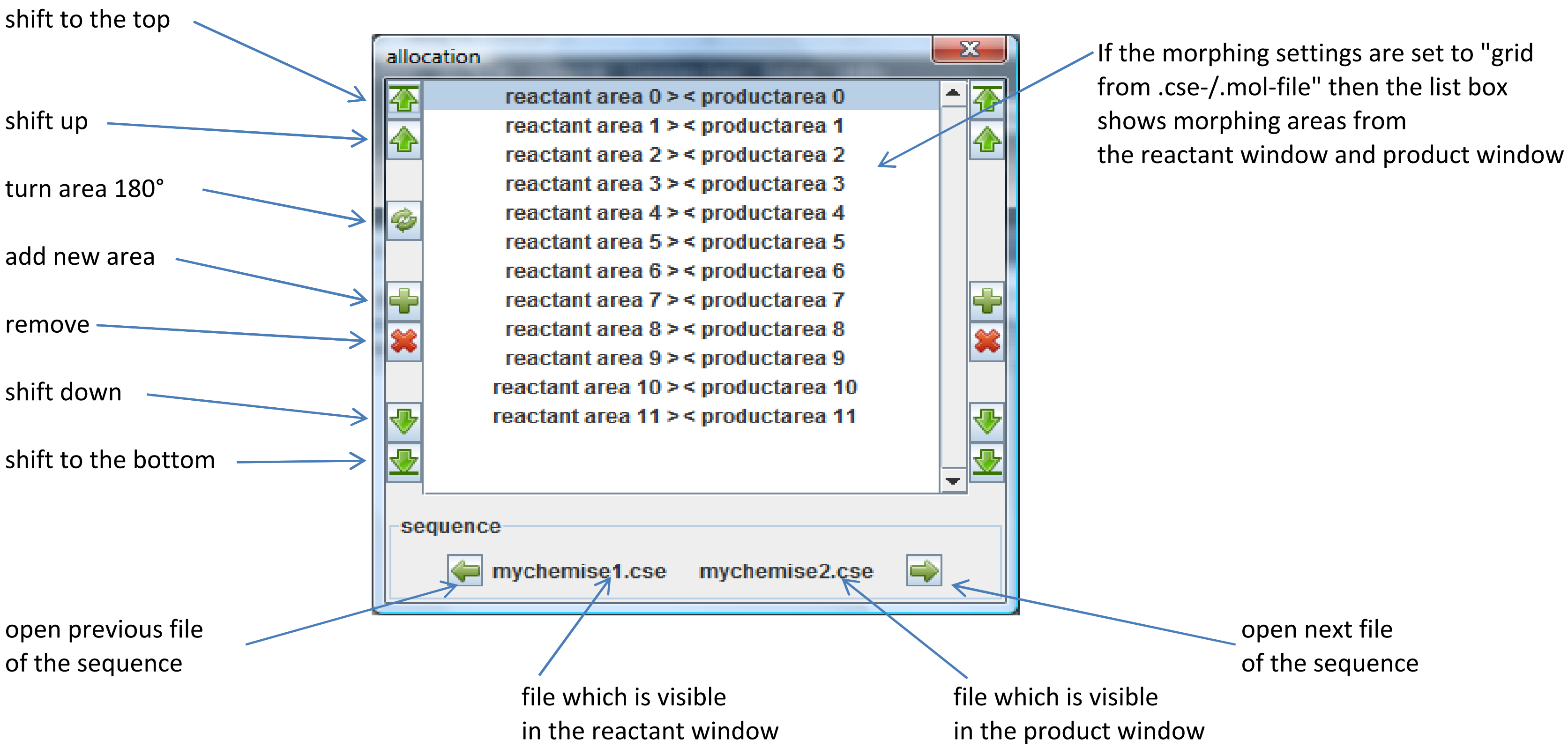

2 Procedure for writing a script

1. Open the script dialog window
2. Drag files into the list box using the mouse
3. Arrange the files in the correct sequence ( )
4. Save as a MyChemise script (.csp)
5. Close the script dialog window; the script toolbar appears
6. Move using buttons of the script-toolbar in the script

   The next page is always opened using

   The contents of the files are shown on the drawing area or in a new window ( ).

   In the new window, you can open the next page by clinking with the mouse.

   MyChemise files (.cse), MyChemise animations (.cai), image files (.jpg, .jpeg, .gif, .png, .bmp) and mol files (.mol) are shown directly in MyChemise.

   Other files (e.g. .xls) are opened using other programs that are registered in the operating system software (e.g. Excel).

8. The script can be run as a slideshow ( )

   The time until the next image is displayed is set using 0 1

3 Making an animation

1. Open the animation dialog window

2. Drag files into the list box using the mouse

3. Arrange the files into the correct sequence ( )

4. Enter the time delay between image changes

5. Save as a MyChemise animation (.cai)

6. Start the animation using start (in most cases it's better to activate endless)
If it is necessary to show the animation in a new window, try it out using new frame or using new frames to see which option gives the best results

5. Close the animation dialog window

6. To end the animation close the window in which it is running or
open the animation dialog window and use stop stop

4 Morphing

You can use morphing to transfer areas of the reactant window stepwise into areas of the product window

1. Open the morphing window 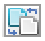
2. Create a new morphing file.

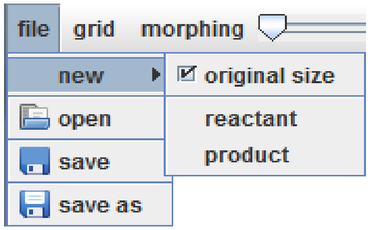

MyChemise files (.cse), image-files (.jpg, .jpeg, .gif, .png, .bmp) and .mol-files. can be used in both the reactant window and product window,
If necessary files can also be opened in their original sizes.
For good morphing effects files of the same size should have already been created (for .cse-files use drawing areas with the same (small!) size).
During morphing every pixel is recalculated. Therefore, the processing speed is dependant on the image size. Therefore set the image size (sketch area) to be as small as possible and not bigger than necessary.

3. Open the set-up (morphing) dialog window.
   Set the numbers of steps (always start with 3 to 5 steps) and the (theoretical) delay time .

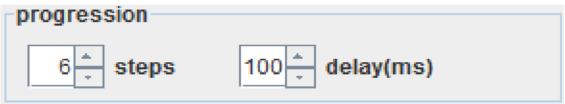

Choose the grid type (for image files - above all 3-point and 4-point mappings and free rectangles are used). Cse and .mol files are directly morphed from 'from cse-file' created grids or 'from free' rectangles.
Also the following applies:
The calculation time needed increases according to the number of areas.

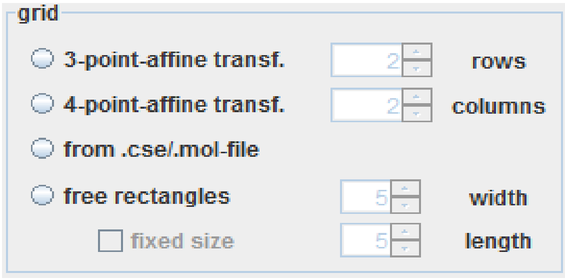

Set-up the behaviour of the areas and that of the background
(areas which are not to be morphed):
- reactant: only what is visible in the reactant window is moved.
- single: as for reactant but in the last step
  that what is visible is shown in the product window.
- morphing: shape and colour are been morphed
- white: white background
- colored: coloured background

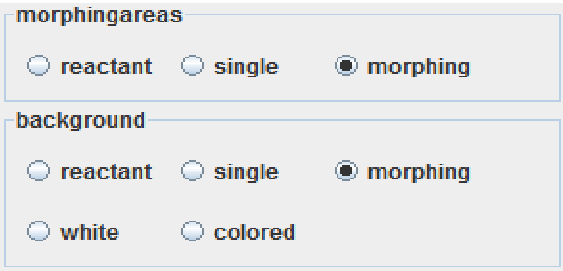

Set-up the time progression:
- single: one pass
- cyclic: pass changes direction at the end, endless
- acyclic: restarts at the beginning again, endless

Close set-up (morphing) dialog window using OK

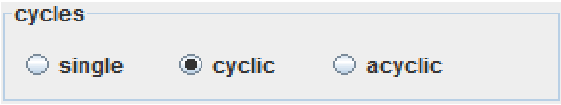

4. For .cse-/.mol-files allocate the areas (for image files carry on from point 5)

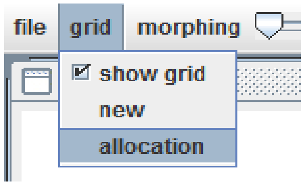

Arrange the files in the right sequence

Open allocation dialog window:
Arrange the reactant and product areas using the arrow buttons 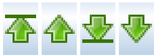 in such a way,
that the areas to be brought together face each other.
Tip: when creating the cse file (sketching the product) by adding and deleting, it is helpful to change the .cse file of the reactant so that the product or an intermediate step is achieved. This prevents those atoms and bonds from being morphed (change of their positions on the screen) that should not really be moved.
Areas can be added or deleted. 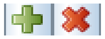
There must be the same number of areas in both the reactant and product windows.

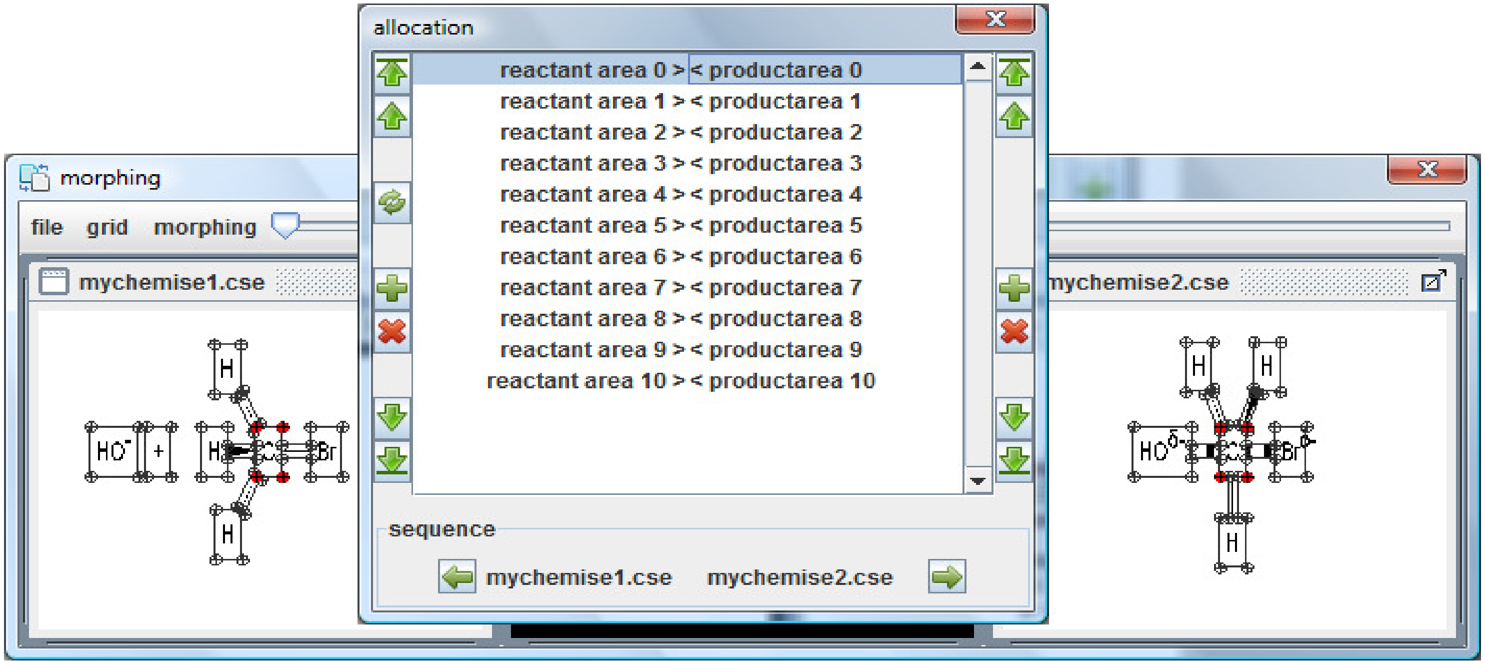

Sometimes an unwanted turning of single areas is visible later during morphing.
These areas then have to be turned. 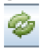
If a morph sequence (-> 4.1) has been created then the names of the predecessor and the follow-up files in the sequence are visible in the lower part of the allocation dialog window. You can open them using the arrow buttons (if .cse files). 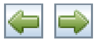
Close the allocation dialog window.

5. Change areas if necessary:
   Clicking in the areas enables you to mark allocated areas in both windows.

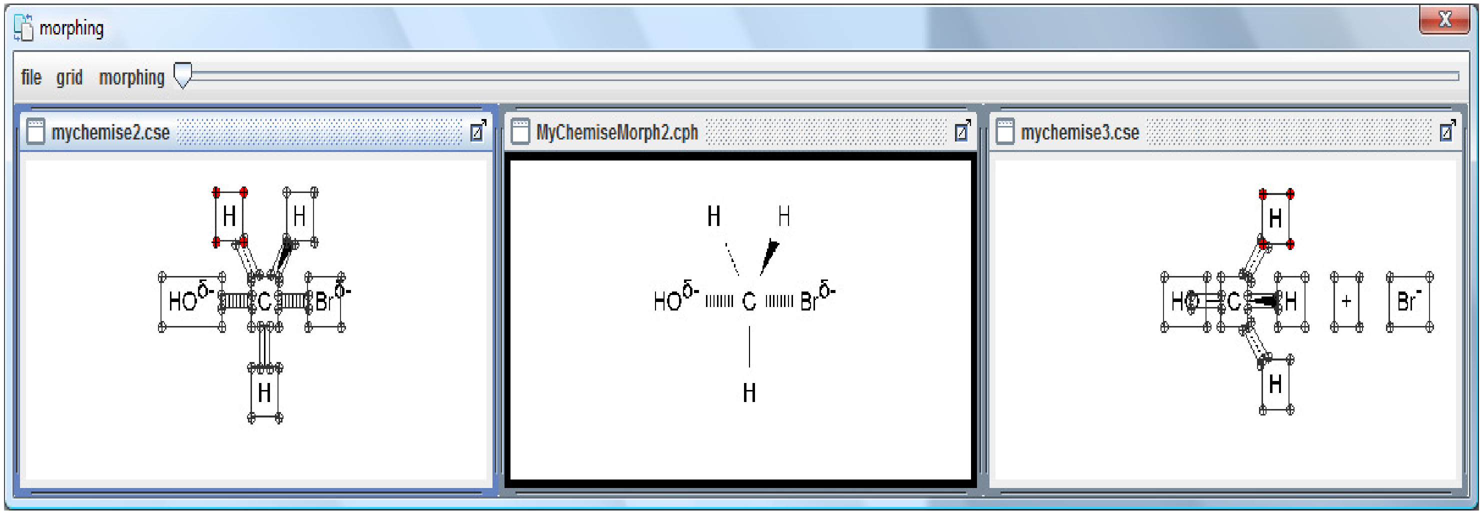

Areas can be moved using a depressed mouse button. If the shift key is also simultaneously held down then the areas in both windows are moved to the same place at same time.
If the alt key is used instad of the shift key the movement is relative to the start position.

The same is valid for single points. Using the ctrl-button it's possible to mark several points at the same time.

In the same manner it is also possible to edit the grids of 3 and 4 point mappings.

For the grid choice 'free rectangles' it is possible to set areas as pairs, in both the reactant and product windows.
Marked areas can be deleted using the Delete key.

6. Save the file

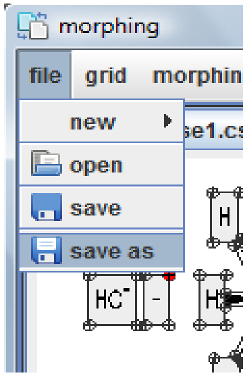

7. Start morphing

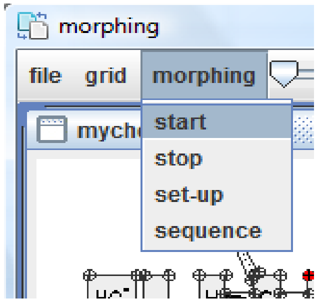

4.1 Making a morphing sequence

Using the mouse, drag the morphing files (.cph), which are to be made into a sequence, into the window.

Top priority is to name the file in which the sequence will be saved (the just opened file; in the example shown on the right MyChemiseMorph1.cph)

If this file is entered at the end again this produces an endless sequence and morphing starts from the beginning again.

Using the arrow buttons, 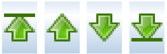 files can be moved to the correct position.

Don't forget to save the file at the end (as a morphing file (.cph)).

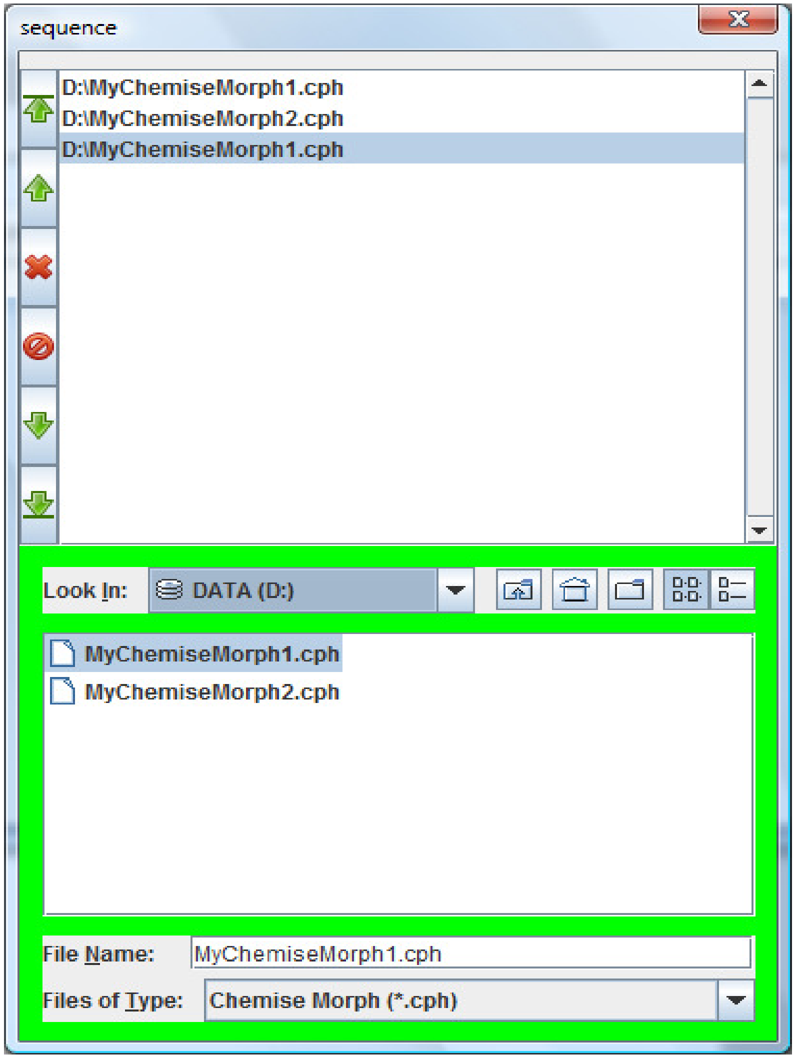

5 Note for Linux users

Linux users experience a problem when copying using the system clipboard.
This problem is well-known for java-programs and is discussed in web forums.
If you want to export a structure as an image into other (text processing) programs, first save your MyChemise file as an image file and then paste the structure directly or with a graphic program into your (text processing) program.
