# Supplementary material for: MyChemise: A 2D drawing program that uses morphing for visualisation purposes
Source: J Cheminform. 2011 Dec 12;3:53. doi: 10.1186/1758-2946-3-53 (PMC3264509; doi:10.1186/1758-2946-3-53)
Supplement: Additional file 2 — mychemise. It contains two files (MyChemise.html and ChemJar.jar). It can be downloaded and installed for running MyChemise in the off-line mode, too. [file 1758-2946-3-53-S2.ZIP › MyChemise.html]

MyChemise
